# Impaired integrin α_5_/β_1_‐mediated hepatocyte growth factor release by stellate cells of the aged liver

**DOI:** 10.1111/acel.13131

**Published:** 2020-03-11

**Authors:** Friederike Rohn, Claus Kordes, Tobias Buschmann, Doreen Reichert, Marianne Wammers, Gereon Poschmann, Kai Stühler, Amelie S. Benk, Fania Geiger, Joachim P. Spatz, Dieter Häussinger

**Affiliations:** ^1^ Clinic of Gastroenterology, Hepatology and Infectious Diseases Heinrich Heine University Düsseldorf Germany; ^2^ Institute for Molecular Medicine Heinrich Heine University Düsseldorf Germany; ^3^ Molecular Proteomics Laboratory BMFZ Heinrich Heine University Düsseldorf Düsseldorf Germany; ^4^ Department of Cellular Biophysics Max‐Planck‐Institute for Medical Research Heidelberg Germany; ^5^ Department of Biophysical Chemistry University of Heidelberg Heidelberg Germany

**Keywords:** aging, hepatic stellate cells, integrins, laminins, mechanobiology

## Abstract

Hepatic blood flow and sinusoidal endothelial fenestration decrease during aging. Consequently, fluid mechanical forces are reduced in the space of Disse where hepatic stellate cells (HSC) have their niche. We provide evidence that integrin α_5_/β_1_ is an important mechanosensor in HSC involved in shear stress‐induced release of hepatocyte growth factor (HGF), an essential inductor of liver regeneration which is impaired during aging. The expression of the integrin subunits α_5_ and β_1_ decreases in liver and HSC from aged rats. CRISPR/Cas9‐mediated integrin α_5_ and β_1_ knockouts in isolated HSC lead to lowered HGF release and impaired cellular adhesion. Fluid mechanical forces increase integrin α_5_ and laminin gene expression whereas integrin β_1_ remains unaffected. In the aged liver, laminin β2 and γ1 protein chains as components of laminin‐521 are lowered. The integrin α_5_ knockout in HSC reduces laminin expression via mechanosensory mechanisms. Culture of HSC on nanostructured surfaces functionalized with laminin‐521 enhances *Hgf* expression in HSC, demonstrating that these ECM proteins are critically involved in HSC function. During aging, HSC acquire a senescence‐associated secretory phenotype and lower their growth factor expression essential for tissue repair. Our findings suggest that impaired mechanosensing via integrin α_5_/β_1_ in HSC contributes to age‐related reduction of ECM and HGF release that could affect liver regeneration.

## Graphical abstract

Hepatic blood flow and sinusoidal endothelial fenestration decrease during aging. Consequently, fluid mechanical forces are reduced in the space of Disse where hepatic stellate cells (HSC) reside. During aging, hepatocyte growth factor (HGF), integrins, and extracellular matrix proteins are significantly reduced in the rat liver and in HSC. The release of HGF by HSC is triggered by mechanical forces via an integrin α5/β1‐mediated mechanism. Thus, less HGF is provided by HSC to support regenerative processes of the aged liver.

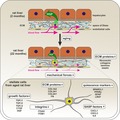

**Abbreviations**
ANOVA: analysis of variance
COL1: collagen 1
COL4: collagen 4
CSF: colony‐stimulating factor
CTGF: connective tissue growth factor
DAPI: 4′,6‐diamidino‐2‐phenylindole
ECM: extracellular matrix
ELISA: enzyme‐linked immunosorbent assay
ELN: elastin
FABP4: fatty acid binding protein 4
FACS: fluorescence‐activated cell sorting
FCS: fetal calf serum
FGF2: fibroblast growth factor 2
FN: fibronectin
GFAP: glial fibrillary acidic protein
GO: gene ontology
gRNA: guide RNA
HGF: hepatocyte growth factor
HRP: horseradish peroxidase
HSC: hepatic stellate cells
IL6: interleukin‐6
IMDM: Iscove's Modified Dulbecco's Medium
ITGA5: integrin α_5_
ITGB1: integrin β_1_
ITS: insulin‐selenite‐transferrin
LAMA2: laminin α2
LAMA4: laminin α4
LAMA5: laminin α5
LAMB1: laminin β1
LAMB2: laminin β2
LAMC1: laminin γ1
LN‐211: laminin‐211
LN‐521: laminin‐521
LRAT: lecithin‐retinol acyltransferase
MMP13: metalloproteinase 13
NGF: nerve growth factor
NID: nidogen
PBS: phosphate buffered saline
PCA: principal component analysis
PEG: polyethylene glycol
PPARγ: peroxisome proliferator‐activated receptor γ
qPCR: quantitative polymerase chain reaction
SASP: senescence‐associated secretory phenotype
SEC: sinusoidal endothelial cells
*SEM*: standard error of the mean
SPARCL1: secreted protein acidic and rich in cysteines‐like 1
TGFb: transforming growth factor β
TLN: talin
TNFR: tumor necrosis factor receptor
VCL: vinculin
α‐SMA: α‐smooth muscle actin

## 1 INTRODUCTION

HSC are liver‐resident mesenchymal stem cells which reside in the space of Disse between sinusoidal endothelial cells (SEC) and hepatocytes. This unique space contains a basement membrane‐like structure composed of laminins and collagen IV (COL4) and serves as a stem cell niche for stellate cells (Kordes & Häussinger, 2013; Kordes, Sawitza, Götze, & Häussinger, 2013; Rohn et al., 2018; Sawitza, Kordes, Reister, & Häussinger, 2009). In chronic liver diseases, HSC become activated and develop into myofibroblast‐like cells which contribute to fibrogenesis (Kisseleva et al., 2012). HSC‐derived myofibroblasts show an increased expression of α‐smooth muscle actin (α‐SMA) and collagen I (COL1) and a downregulation of quiescence markers such as glial fibrillary acidic protein (GFAP; Gard, White, & Dutton, 1985). The activation of HSC is reversible as demonstrated in fibrotic liver and culture of HSC‐derived myofibroblast‐like cells on laminin‐521 (LN‐521; Kisseleva et al., 2012; Rohn et al., 2018). HSC fulfill important functions as mesenchymal stem cells during liver homeostasis and regeneration (Kordes, Sawitza, Götze, Schumacher, & Häussinger, 2015). One function is the release of trophic factors such as hepatocyte growth factor (HGF) which maintains hepatocyte functions and induces their proliferation after liver injury to facilitate tissue repair (Stolz, Mars, Petersen, Kim, & Michalopoulos, 1999; Sumii et al., 2016).

Characteristics of quiescent HSC are maintained in their perivascular niche, the space of Disse. Here, ECM components such as LN‐521 are involved in HSC maintenance (Rohn et al., 2018). The heterotrimeric laminin chains LN‐521 and LN‐511 are important to maintain stem cell characteristics (Domogatskaya, Rodin, Boutaud, & Tryggvason, 2008; Rodin, Antonsson, Hovatta, & Tryggvason, 2014) and are synthesized by pericytes (Jeon et al., 1996) and endothelial cells as reported for the kidney, cornea, skin, and lymph nodes (Miner, 2012; Okumura et al., 2015; Sorokin et al., 1997). In contrast to ECM remodeling during regeneration of injured or diseased liver (Kim, Mars, Stolz, Petersen, & Michalopoulos, 1997), little is known about age‐related changes of hepatic ECM. Although liver function is fairly stable during aging (Kitani, 1991), there are some age‐related alterations in liver physiology such as a reduction of blood flow by 20%–55% and a rarefication of endothelial fenestration (Le Couteur & McLean, 1998; McLean et al., 2003) suggesting that fluid mechanical forces are reduced in the space of Disse during aging.

Biondo‐Simões Mde et al. (2006) provided evidence that liver regeneration after partial hepatectomy (PHx) is delayed in old rats. This observation was supported by additional studies demonstrating an impaired regenerative potential of the aged liver in rodents and humans (Enkhbold et al., 2015; Zhu et al., 2014). A possible explanation could be the significant reduction of the HGF amount in the aged liver (Enkhbold et al., 2015; Zhu et al., 2014). However, the reasons for impaired liver regeneration are not fully understood. Only few reports are available about the behavior of HSC during aging thus far, although HSC are important players in liver regeneration (Kordes et al., 2015). The abundance of HSC and the number as well as size of their retinoid‐containing lipid droplets seems to increase during aging, but contradicting results have been reported about their activation state (Maeso‐Díaz et al., 2018; Warren et al., 2011). Moreover, the impact of aging on HSC function and the underlying mechanisms have not been addressed so far. Therefore, the present study was conducted to elucidate age‐related alterations in HSC and their perivascular niche and consequences for the regenerative potential of the aged liver.

## 2 RESULTS

### 2.1 Altered ECM composition in aged rat liver

**Figure 1:**
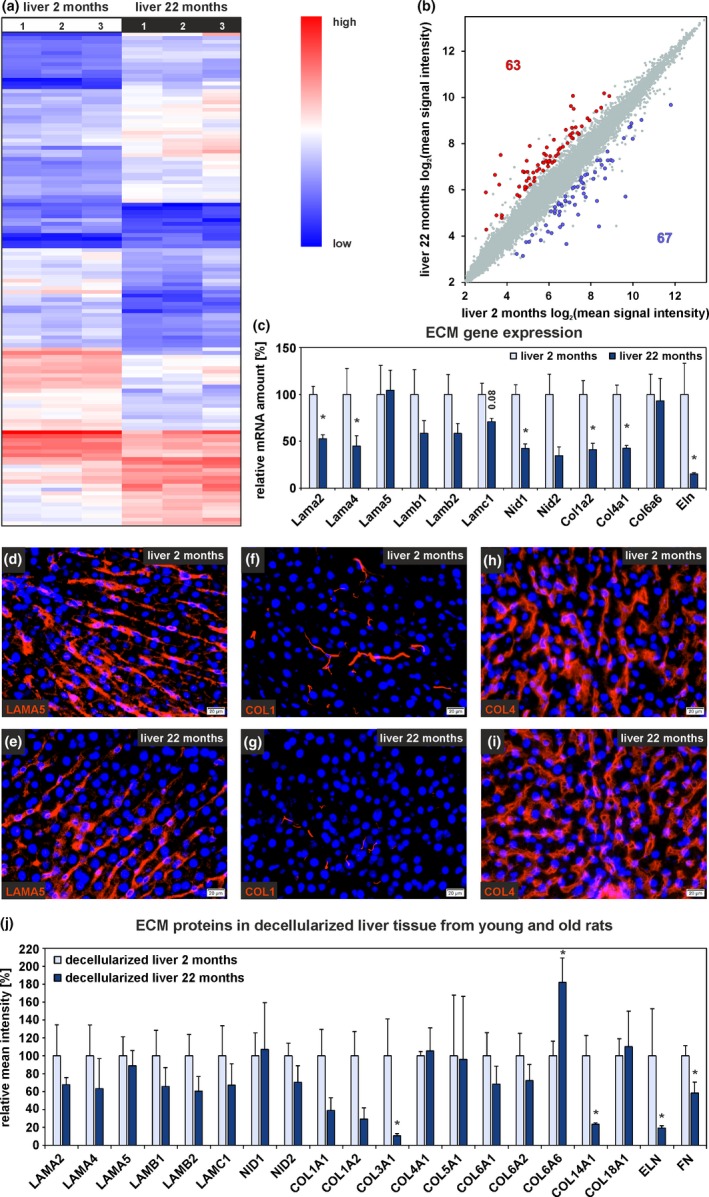
Altered ECM protein gene expression in liver tissue during aging. (a) Hierarchical cluster analysis of differentially expressed genes in whole liver tissue from 2‐month‐old and 22‐month‐old rats. (b) Scatter plot of gene expression of whole liver tissue from both age groups (*n* = 3 per age group, fold change > 2, *p* < .05, ANOVA). (c) Expression analyses of genes encoding for ECM proteins by qPCR. Mean expression of liver tissue samples from young rats was set to 100% (*n* = 8–10 for 2‐month‐old and *n* = 8–9 for 22‐month‐old rats, *p < .05). Immunofluorescence analysis of liver tissue sections from 2‐month‐old and 22‐month‐old rats (*n* = 3) with antibodies against (d, e) LAMA5, (f, g) COL1, and (h, i) COL4 (red). Cell nuclei were stained with DAPI (blue; scale bars: 20 µm). (j) Proteome analysis of decellularized rat liver tissue. Mean intensities of samples from young rats were set to 100% (*n* = 3, *p < .05). (c, j) Data are presented as means ± *SEM*

**Figure 2:**
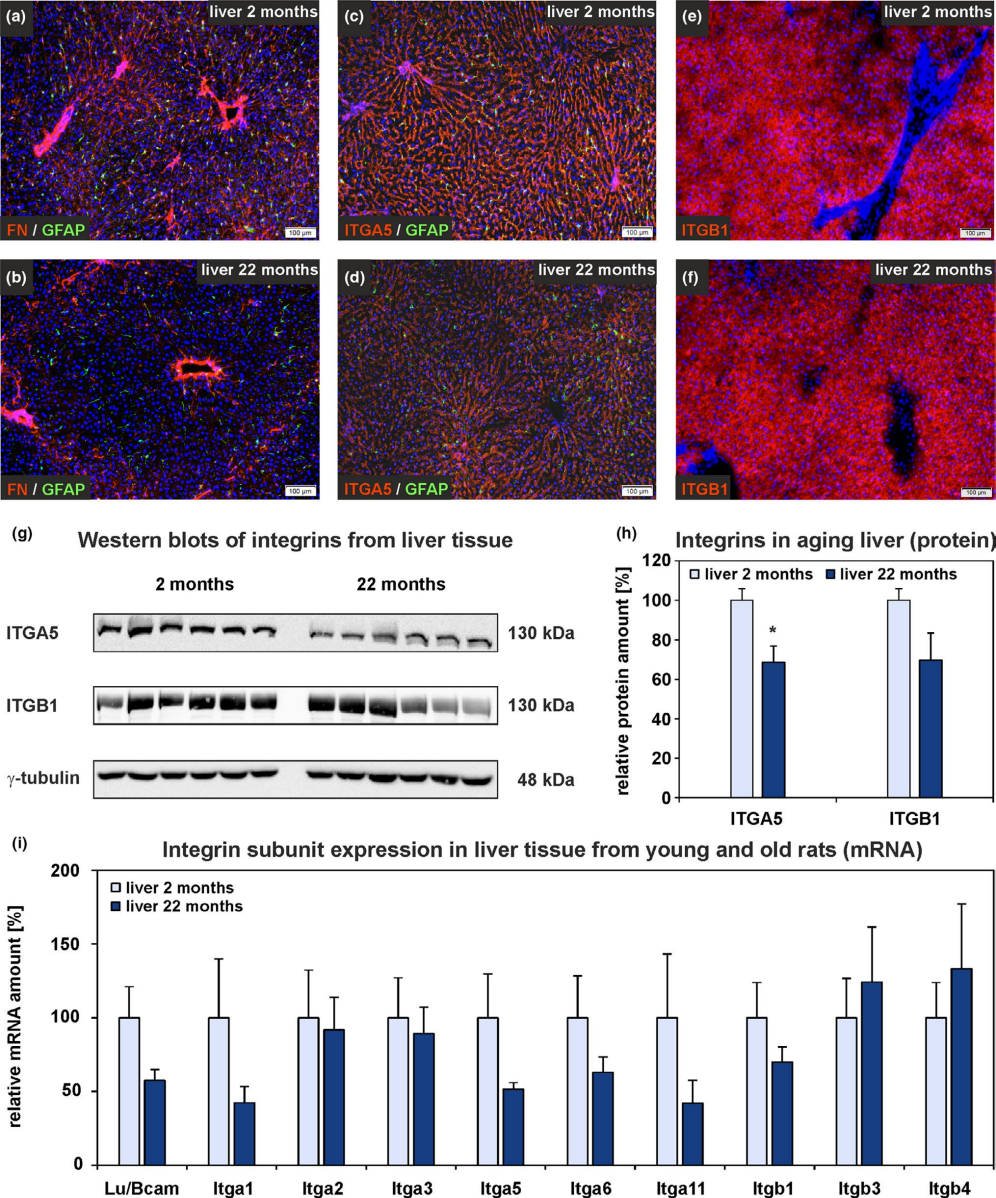
FN and integrin subunits in aged rat liver. (a–f) Immunofluorescence analysis of liver sections from 2‐month‐old and 22‐month‐old rats with antibodies against (a, b) FN (red) and (c, d) ITGA5 (red) in combination with the stellate cell marker GFAP (green). (e, f) Immunofluorescence of ITGB1 (red) of young and old rat liver. Cell nuclei were stained with DAPI (blue; scale bars: 100 µm). (g) Western blot analyses of whole liver samples from young and old rats using antibodies against ITGA5 and ITGB1. γ‐tubulin served as loading control. (h) Densitometric analysis of ITGA5 and ITGB1 Western blots. Values of young rats were set to 100% (*n* = 6 for both age groups; *p < .05). (i) qPCR analyses of Lutheran blood group/basal cell adhesion molecule (*Lu/Bcam*) and integrin subunit expression in old compared to young rat liver tissue. Mean expression of liver tissue samples from young rats was set to 100% (*n* = 8–10, *p < .05). (h, i) Data are presented as means ± *SEM*

Whole liver tissue from young (2 months) and old (22 months) rats was analyzed by gene expression microarrays, qPCR, immunofluorescence, and Western blot (Figure S1A). The microarray raw data exhibited equal signal intensities and no obvious differences in Pearson correlation analysis between age groups (Figure S2A,C). Principal component analysis (PCA) revealed that liver samples from both age groups could be separated into distinct groups (Figure S2E). Hierarchical cluster analysis of microarray data of liver tissue showed a differential expression of 130 genes out of 30,429 analyzed genes (Figure 1a). In detail, 63 genes were significantly upregulated (Figure 1b, red dots) while 67 genes were significantly downregulated in liver tissue from old compared to young rats (Figure 1b, blue dots). All significantly altered genes associated with the ECM were found to be downregulated (Table S1). ECM‐associated gene expression such as laminin α2 (*Lama2*), *Lama4*, nidogen 1 (*Nid1*), collagen 1α2 (*Col1a2*), *Col4a1,* and elastin (*Eln*) was investigated in detail by qPCR using additional samples. The expression of these genes was significantly reduced in liver tissue from old rats while the expression of *Lama5* remained unchanged and laminin β1 (*Lamb1*), *Lamb2*, laminin γ1 (*Lamc1*), and *Nid2* tended to decrease without reaching significance (Figure 1c). Immunofluorescence staining of liver sections with antibodies against LAMA5, collagen 1 (COL1), and collagen 4 (COL4) revealed no obvious differences in fluorescence signal intensities of different age groups (Figure 1d–i). To further quantify protein composition changes of hepatic ECM during aging, a proteome analysis of decellularized liver tissue from young and old rats was performed (Figure S1B). The protein amount of different laminin chains (LAMA2, LAMA4, LAMB2, LAMC1), NID2, and various collagens (COL1A1, COL1A2, COL3A1, COL6A1, COL6A2, COL14A1) supported the mRNA data and declined in hepatic ECM of old rats (Figure 1j). Furthermore, ELN and FN showed a significant reduction in the decellularized livers of 22 months compared to 2‐month‐old rats. In contrast, COL4A2, COL4A5, and COL6A5 amounts were elevated, but without reaching significance (Table S2). Only COL6A6 increased significantly in old rat liver as determined by proteome analysis (Figure 1j). FN in liver sections from old rats was found to persist around larger vessels but clearly declined in the remaining parenchyma as indicated by immunofluorescence of liver sections (Figure 2a,b). Since FN was markedly reduced in hepatic ECM from old rats, the subunits of the FN receptor integrin α_5_/β_1_ (ITGA5 and ITGB1) were investigated in whole liver tissue from young and old rats by immunofluorescence and Western blot (Figure 2c–h). In line with the microarray data (Table S1), also the protein amount of ITGA5 significantly declined during aging by 30% whereas ITGB1 showed a trend toward reduction (Figure 2h). Among integrins, *Itga1*, *Itga5*, *Itga6*, *Itga11*, and *Itgb1* tended to decrease at the mRNA level in the aged liver (Table S1, Figure 2i).

### 2.2 Impaired HSC characteristics in the aged rat liver

**Figure 3:**
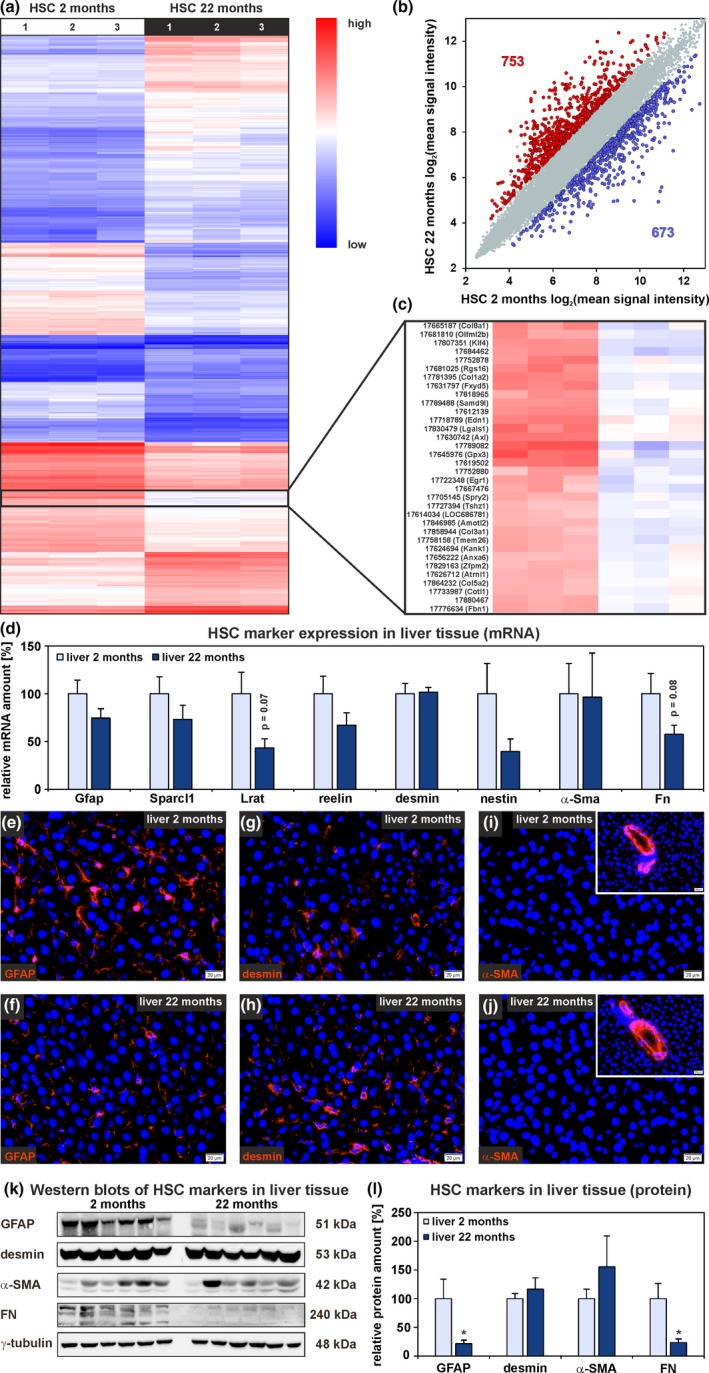
Alterations of HSC characteristics during aging. (a) Hierarchical cluster analysis of differential expressed genes in HSC from 2‐month‐old and 22‐month‐old rats. (b) Scatter plot of gene expression in HSC from young and old rats (*n* = 3 per age group, fold change > 2, *p* < .05, ANOVA). (c) The expression of different collagens (*Col8a1*, *Col1a2*, *Col3a1*, and *Col5a2*) decreased in HSC from old rats. (d) Expression analyses by qPCR of genes typically observed in quiescent and activated HSC using whole liver samples from both age groups. Immunofluorescence analysis of liver tissue sections (red) with antibodies against (e, f) GFAP, (g, h) desmin, and (i, j) α‐SMA (inserts: α‐SMA at blood vessels). Cell nuclei were stained by DAPI (blue; *n* = 3; scale bars: 20 µm). (k, l) Western blot analyses of markers typically observed in quiescent and activated HSC in liver tissue from young and old rats. (d, l) Mean values of liver tissue samples from young rats were set to 100%. Data are presented as means ± *SEM* (*n* = 10 for 2‐month‐old rats, *n* = 9 for 22‐month‐old rats, *p < .05)

HSC isolated from old (22 months) rats showed a stronger vitamin A signal during FACS than HSC from young (2 months) rats (Figure S1D). After one day of culture, sorted HSC were collected for analysis by gene expression microarrays. The microarray data exhibited equal signal intensities within each group (Figure S2B), and Pearson correlation analysis revealed that HSC from old rats were different from those of young (Figure S2D). The three HSC samples of each age group clustered together when a PCA was performed (Figure S2F). Gene expression microarrays of HSC from young and old rats revealed a differential expression of 1,426 genes (Figure 3a). The expression of 753 genes significantly increased (Figure 3b, red dots) and 673 genes significantly decreased in HSC from 22‐month‐old rats (Figure 3b, blue dots). Interestingly, genes encoding for different collagens such as *Col8a1*, *Col1a2*, *Col3a1*, and *Col5a1* showed lower expression in HSC from old rats (Figure 3c, Table S3). In line with this, expression of further genes associated with the ECM such as *Lama2*, *Lamc1*, *Nid1*, *Nid2*, and *Fn1* was significantly downregulated in HSC from old rats (Table S3). GO term analysis of significantly up‐ and downregulated genes revealed that biological processes associated with development, differentiation, aging, inflammation, cell death, and cell migration as well as extracellular matrix organization, cell adhesion, and integrin‐mediated signaling pathway appeared in the set of significantly altered genes in HSC from old rats (Figure S3, Table S4). Although typical markers of activated HSC such as collagens decreased and *α‐Sma* remained unchanged during aging as indicated by microarrays, the quiescence marker secreted protein acidic and rich in cysteines‐like 1 (*Sparcl1*) decreased (Table S5) and expression of genes associated with ECM remodeling and cell migration such as matrix metalloproteinase 13 (*Mmp13*; Table S3) and *Cxcr4* (Table S6) increased, suggestive for impaired HSC maintenance. In addition, focal adhesion elements such as vinculin (*Vcl*) and talin (*Tln1*, *Tln2*) were significantly decreased in HSC from old rats (http://www.ebi.ac.uk/arrayexpress; accession number: E‐MTAB‐7423) indicating impaired anchorage of HSC in their stem cell niche.

Additional markers associated with HSC quiescence and activation were analyzed in further liver tissue samples from young and old rats. The results from qPCR analysis demonstrated reduced expression of the genes *Gfap*, lecithin‐retinol acyltransferase (*Lrat*), reelin, nestin, and *Fn* in livers from aged rats, while desmin and *α‐Sma* showed no differential expression (Figure 3d). Immunofluorescence of liver tissue sections and Western blot analyses supported qPCR data and showed weaker fluorescence intensities and significantly reduced protein amounts of GFAP and FN in old rat liver (Figures 2a,b and 3e,f,k,l). No differences in protein levels were detected for desmin and α‐SMA by immunofluorescence and Western blot (Figure 3g–l). These observations indicated a loss of original HSC characteristics during aging without synthesis of FN, fibrillar collagens, and α‐SMA as known for HSC‐derived myofibroblast‐like cells in fibrotic processes.

### 2.3 Senescence‐associated secretory phenotype of HSC from aged rats

Many senescence‐associated secretory phenotype (SASP) factors were significantly upregulated in the gene expression microarrays of HSC from old rats (Table S6). Among them, multiple factors involved in inflammatory processes such as interleukin 1α (*Il1a*), *Il6*, tumor necrosis factor receptors (*Tnfrsf1a/b*), and colony‐stimulating factors (*Csf2/3*) as typical elements of SASP were found (Table S6).

In contrast, the expression of growth factors such as transforming growth factor β (*Tgfb1*, *Tgfb2*, *Tgfb3*), fibroblast growth factor 2 (*Fgf2*), connective tissue growth factor (*Ctgf*), and *Hgf* significantly decreased in HSC during aging. However, nerve growth factor (*Ngf*) showed elevated expression in HSC from aged rats (Table S6).

A rat cytokine antibody array was used to quantify cytokines in blood serum and culture supernatants of isolated HSC from young and old rats (Figure S1C,D). No marked differences in cytokine concentrations of the sera were measured with exception of CXCL7, which was significantly reduced in samples from old rats (Figure S4A). The sensitivity of the array was quite low when culture supernatants of HSC from young and old rats were analyzed and only CXCL1 and TIMP‐1 could be detected in all samples. While TIMP‐1 remained unchanged, CXCL1 protein levels increased in culture supernatants of HSC from aged rats (Figure S4B,C). In addition to protein arrays, ELISA analyses of CXCL3 and IL6 were performed to investigate additional SASP factors at the protein level. Both cytokines were markedly elevated in conditioned culture medium of HSC from old compared to young rats (Figure S4D,E). These results were in accordance with the data obtained by microarray analysis and indicated cellular senescence in HSC from old rats (Table S6).

### 2.4 Influence of mechanical forces on HSC function

**Figure 4:**
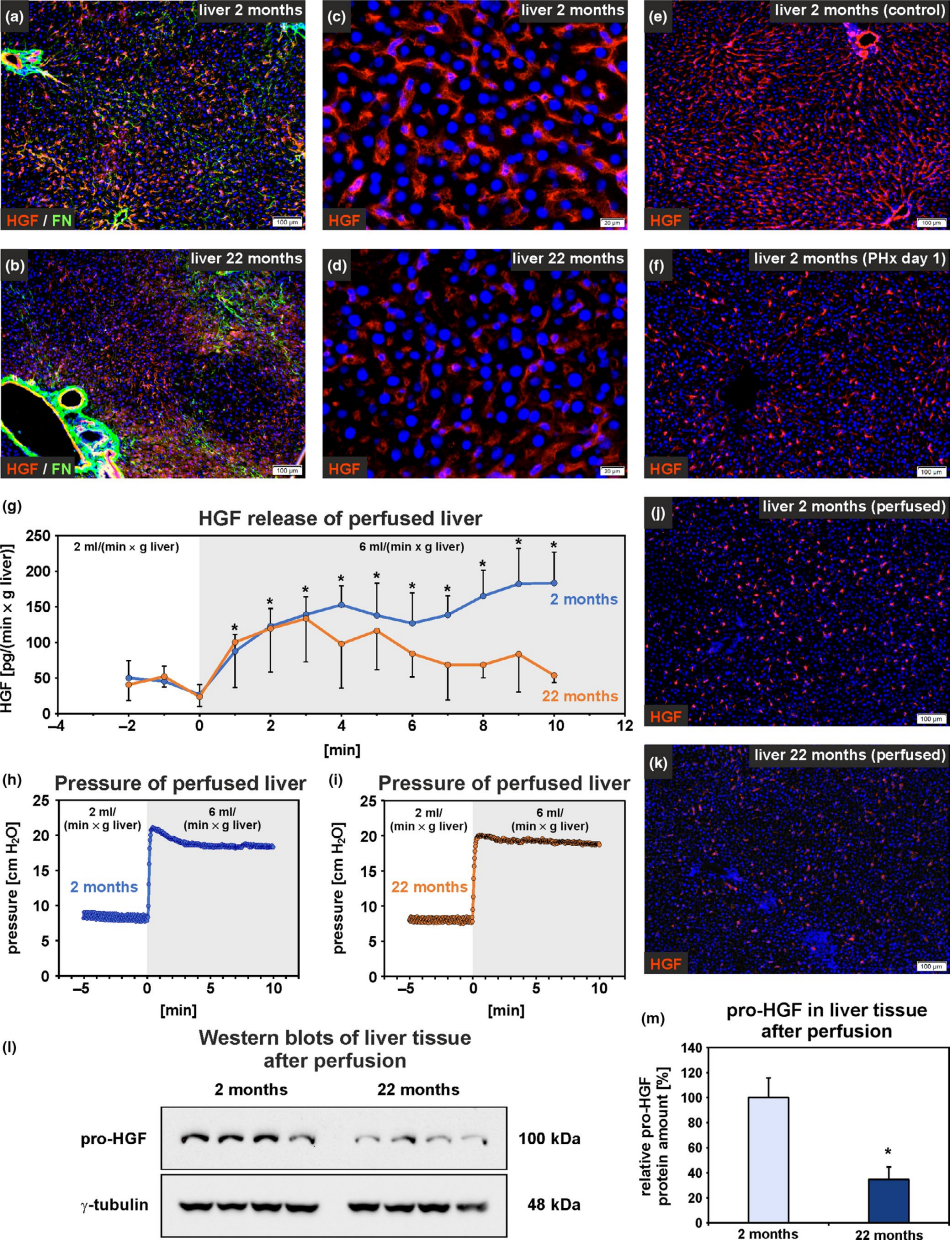
HGF deposition and release decrease in the aged liver. (a, b) HGF (red) was detected in liver sinusoids by immunofluorescence where it was co‐localized with FN (green; scale bars: 100 µm). (c, d) The HGF fluorescence intensity was reduced in old compared to young rat liver (scale bars: 20 µm). HGF (red) in liver sinusoids of the young rat liver (e) without and (f) with partial hepatectomy (PHx). One day after PHx, HGF was reduced in liver sinusoids. The cell nuclei are stained by DAPI (blue; *n* = 3; scale bars: 100 µm). (g–m) Mechanical stimulation of liver tissue by elevated perfusion rates. (g) The liver from young and old rats were perfused via the portal vein using initially 2 ml/(min × g) liver weight. The flow rate was then elevated to 6 ml/(min × g liver weight) at the time point 0 min. HGF was quantified in the effluent by ELISA. Statistical comparison of the HGF values before and after raising the flow rate within one age group (*n* = 3–4; *p < .05). (h, i) Young and old rat liver was exposed to comparable liquid pressures, which were continuously recorded during perfusion as shown here for two individual livers. Immunofluorescence of HGF using liver sections from (j) young and (k) old rats 30 min after increasing the perfusion rate in comparison with mechanically unstimulated liver shown in c/e and d. (l, m) Quantification of the remaining pro‐HGF in whole liver tissue by Western blotting after elevation of the perfusion rate for 30 min (*n* = 4). (m) Densitometric analysis of the pro‐HGF protein bands shown in (l) revealed lower residual pro‐HGF levels in old compared to young rat liver (*p < .05). (g, m) Data are presented as means ± *SEM*

Immunofluorescence staining of rat liver sections revealed that the HGF protein was present in liver sinusoids where it co‐localized with FN (Figure 4a,b). Similar to FN, the intensity of HGF immunofluorescence was reduced in liver tissue of old compared to young rats (Figure 4a–d). Western blot analyses of whole liver tissue from young and old rats revealed a reduction of the matrix‐bound pro‐HGF protein amount by 62 ± 10% (*n* = 6) in aged liver tissue (Figure S5). After partial hepatectomy (PHx), which is associated with increased HGF release and proliferation of hepatocytes (Lindroos, Zarnegar, & Michalopoulos, 1991), the HGF immunofluorescence intensity significantly decreased by more than 26 ± 4% (*n* = 3) in liver sinusoids as demonstrated here for young rats one day after PHx (Figure 4e,f). At this time point, hepatocytes showed maximal proliferation as investigated by pulse labeling of DNA with bromodeoxyuridine (25 mg/kg body weight; 4 hr) in proliferating cells (not shown).

Since hepatic blood flow is increased shortly after PHx but reduced during aging (Le Couteur & McLean, 1998; Lorenz et al., 2018), it was investigated whether mechanical forces such as fluid shear stress and stretching trigger the HGF release in liver perfusion experiments. The liver tissues of both age groups were perfused via the portal vein with Krebs–Henseleit buffer and exposed to increased mechanical forces by rising the flow rate (Figure 4g–m). Significantly elevated HGF release into the effluent was measured for young rat liver after applying mechanical forces via the perfusion buffer, whereas statistical significance was not achieved for aged rat liver when HGF values before and after increasing the flow rate were compared (Figure 4g). Similar to the situation after PHx, HGF was found to be reduced in the liver sinusoids by the elevated perfusion rate (Figure 4e,f,j,k) and the remaining pro‐HGF amount was significantly lower in whole liver samples of aged compared to young rats as investigated by Western blot (difference of 65%; Figure 4l,m).

**Figure 5:**
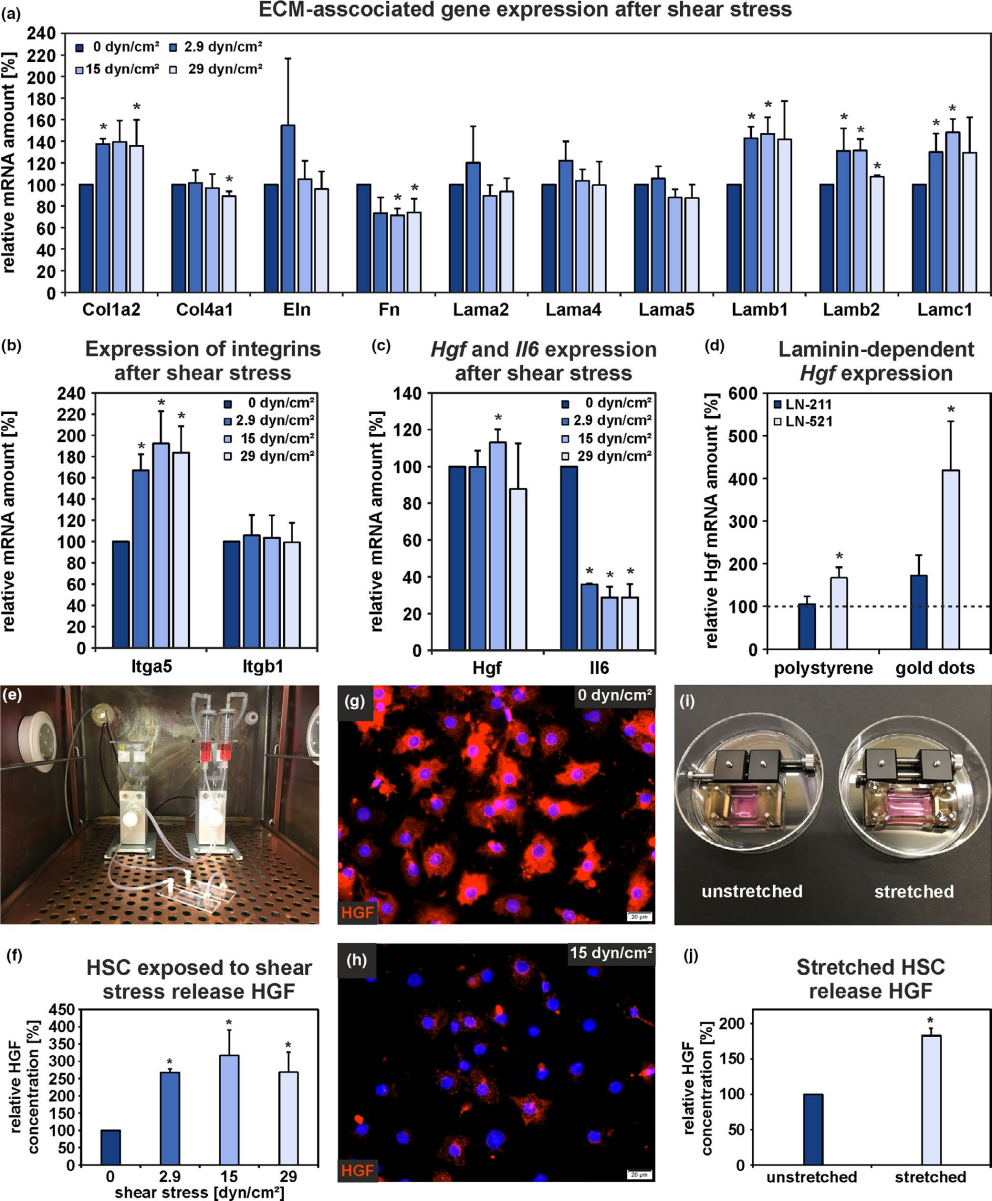
Mechanical forces stimulate HGF release from HSC. Isolated HSC were exposed to either shear stress or stretching to investigate changes in gene expression and HGF release in comparison to mechanically unstimulated cells. (a) Expression analyses by qPCR of ECM‐associated genes, (b) *Itga5* and *Itgb1*, and (c) *Hgf* and *Il6* using RNA samples obtained from HSC exposed to fluid shear stress for 1 hr. The mRNA expression of HSC under static condition (0 dyn/cm^2^) was set to 100% for each independent experiment (*n* = 3; *p < .05 for shear stress vs. static condition). (d) *Hgf* expression analyses by qPCR in HSC cultured for 7 days under serum‐free conditions on uncoated or laminin‐coated (LN‐211, LN‐521) culture surfaces. Polystyrene and nanostructured surfaces with a spacing of 150 nm between gold dots were used. The mRNA expression of HSC cultured for one week on uncoated polystyrene dishes was set to 100% (broken line) for each experiment (*n* = 5; *p < .05). HGF release by HSC exposed to (e–h) fluid shear stress or (i, j) mechanical stretching as analyzed by ELISA and immunofluorescence. HGF concentration of culture supernatants from static condition (0 dyn/cm^2^) and unstretched HSC were set to 100% for each independent experiment (*n* = 4; *p < .05). Immunofluorescence analysis of HGF (red) in HSC showed a high fluorescence signal intensity under (g) static condition, (h) which was lost under fluid shear stress for 1 hr. Cell nuclei are stained by DAPI (blue; scale bars: 20 µm). (a–d, f, j) Data are presented as means ± *SEM*

It was also investigated whether fluid shear stress and stretching influence HSC function (Figure 5a–j). Therefore, freshly isolated HSC were exposed to low (2.9 dyn/cm^2^), medium (15 dyn/cm^2^), or high (29 dyn/cm^2^) shear stress under laminar pulsatile flow (2.5 Hz) and compared to HSC under static conditions (0 dyn/cm^2^). Interestingly, shear stress significantly upregulated the expression of *Col1a2*, *Lamb1*, *Lamb2*, *Lamc1*, and *Itga5* in HSC (Figure 5a,b), genes that exhibited decreased expression in HSC and liver tissue during aging (Figures 1c and 2i; Tables S1 and S3). Although FN mRNA and protein were significantly decreased in HSC and whole liver tissue during aging, the expression of *Fn* showed a slight but in case of 15 and 29 dyn/cm^2^ significant downregulation in HSC indicating additional regulatory mechanisms for FN (Figure 5a). While the expression of *Itga5* was significantly increased by about 80% by shear stress, *Itgb1* expression was not affected (Figure 5b). However, *Itgb1* was already highly expressed under static condition (0 dyn/cm^2^). In contrast to its reduced expression in HSC and liver tissue from aged rats, *Itga6* expression was not affected by shear stress (not shown). The *Hgf* expression was only slightly influenced, whereas *Il6* expression, which was significantly increased in HSC from old rats, was downregulated by about 65% under all shear stress conditions (Figure 5c).

The expression of *Hgf* was mainly upregulated by laminin chains which were significantly reduced in the rat liver matrix during aging and which could be induced by shear stress (*Lamb1*, *Lamb2*, *Lamc1*). HSC cultured on either laminin‐521 (LN‐521)‐coated polystyrene or on LN‐521‐functionalized nanostructured surfaces comprising of gold dots with a defined spacing (150 nm) for one week showed significantly increased *Hgf* expression compared to HSC cultured on uncoated polystyrene (Figure 5d). Moreover, a defined distance between the single LN‐521 molecules further elevated the *Hgf* mRNA amount. In contrast, HSC cultured on LN‐211‐coated surfaces showed no altered *Hgf* expression compared to cells on polystyrene (Figure 5d). Additionally, qPCR analysis of HSC cultured for seven days on either polystyrene uncoated or coated with COL4 or FN revealed no significant alterations in *Hgf* expression but tended to increase on FN by about 45% (Figure S6a). Furthermore, HGF release by HSC into the culture medium was investigated by ELISA and revealed a significantly higher HGF concentration in the supernatants from cells on LN‐521, COL4, and FN compared to cells on LN‐211 (Figure S6b). Alterations in *Hgf* expression and HGF release indicate that the ECM composition critically influences HSC function.

HGF release was analyzed after applying shear stress and stretching (Figure 5e–j). Under both conditions, HSC released significantly more HGF into the culture medium compared to HSC under static and unstretched conditions (Figure 5f,j). Immunofluorescence analysis of HGF before and after shear stress supported the ELISA data and showed a low fluorescence signal intensity in HSC after applying mechanical forces (Figure 5g,h).

**Figure 6:**
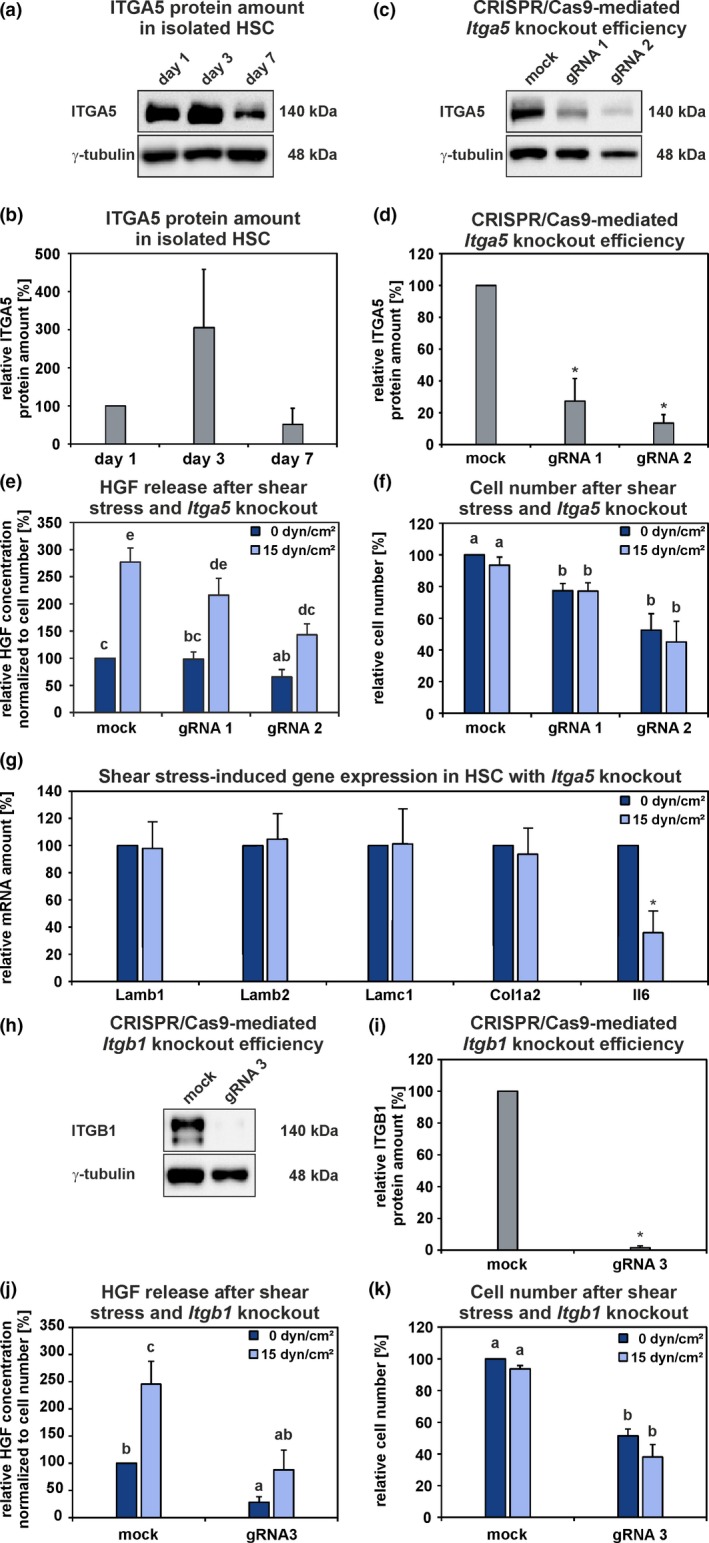
Influence of shear stress on HSC with CRISPR/Cas9‐mediated *Itga5* or *Itgb1* knockout. (a, b) ITGA5 was detectable in freshly isolated (day 1) and culture‐activated (day 3 and 7) HSC by Western blot analysis. The ITGA5 protein amount in HSC at day 1 of culture was set to 100% for each independent experiment (*n* = 3). (c, d) CRISPR/Cas9‐mediated *Itga5* knockout in HSC was evaluated by Western blot. The efficiencies of two different gRNA was assessed in comparison to the ITGA5 protein amount of the control (mock; 100%) and γ‐tubulin served as a loading control (*n* = 3; *p < .05). (e) HSC with *Itga5* knockout were exposed to fluid shear stress (15 dyn/cm^2^) for 1 hr and the HGF concentration of the culture supernatant was measured by ELISA. The data were normalized to cell number. The HGF concentration from mock static condition was set to 100% for each independent experiment (*n* = 5; a, b, c, d, e; *p* < .05; groups sharing the same letters did not differ significantly). (f) Number of adherent HSC before and after shear stress experiments. The cell number from mock static condition was set to 100% for each independent experiment (*n* = 5; a, b; *p* < .05). (g) Expression analyses of ECM‐associated genes and *Il6* in HSC with *Itga5* knockout (gRNA 1) before and after shear stress was applied. The mRNA amount of the static condition was set to 100% for each independent experiment (*n* = 3; *p < .05). The mRNA yield obtained from HSC with *Itga5* knockout using the gRNA 2 was too low for qPCR‐based expression analysis. ITGB1 is not detectable in quiescent HSC but appears early during their activation (Kordes et al., 2013). (h, i) CRISPR/Cas9‐mediated Itgb1 knockout in HSC was evaluated by Western blot. The efficiency of gRNA 3 was determined in comparison to the ITGB1 protein amount of the control (mock; 100%) and γ‐tubulin served as a loading control (*n* = 3; *p < .05). (j) HSC with *Itgb1* knockout were exposed to fluid shear stress (15 dyn/cm^2^) for 1 hr and the HGF concentration of the culture supernatant was measured by ELISA. The data were normalized to cell number. The HGF concentration from mock static condition was set to 100% for each independent experiment (*n* = 3; a, b, c; *p* < .05). (k) Number of adherent HSC before and after shear stress experiments. The cell number from mock static condition was set to 100% for each independent experiment (*n* = 3; a, b; *p* < .05). The mRNA yield obtained from HSC with *Itgb1* knockout was too low for qPCR‐based expression analysis. (b, d, e–g, i–k) The data are indicated as means ± *SEM*

Interestingly, ITGA5 was significantly reduced in HSC and liver tissue from old rats (Figure 2c,d,g,h; Tables S1 and S3) and its expression could be induced when HSC were exposed to shear stress for one hour (Figure 4b). Western blot analyses of freshly isolated HSC indicated that ITGA5 is already present in their quiescent state (Figure 6a,b). To investigate the relevance of ITGA5 as a possible mechanosensor in HSC, this integrin subunit was knocked out by CRISPR/Cas9 using two guide RNA (gRNA), which showed different knockout efficiencies of about 73% (gRNA 1) and 87% (gRNA 2), respectively (Figure 6c,d). HSC that underwent *Itga5* knockout exhibited lowered HGF release already under static conditions when the control (mock) was compared with HSC that received gRNA 2 (Figure 6e). However, a stimulation of HGF release after applying shear stress (15 dyn/cm^2^) was still detectable in HSC after *Itga5* knockout, but the total amount of HGF released after shear stress decreased markedly with increasing knockout efficiency (Figure 6e). These data suggest the involvement of ITGA5 in HGF release by HSC. Furthermore, HSC without *Itga5* adhered less efficiently to the culture surface compared to control (Figure 6f). Finally, the expression of genes which could be induced by shear stress (Figure 4a) was analyzed by qPCR. Interestingly, the expression of ECM‐associated genes (*Lamb1*, *Lamb2*, *Lamc1*, *Col1a2*) was not altered by shear stress in HSC with *Itga5* knockout while the expression of *Il6* was still significantly reduced (Figure 6g) indicating that ITGA5 is involved in the mechanosensing of HSC. In contrast to ITGA5, ITGB1 protein is not detectable in quiescent HSC but appears early during activation as demonstrated previously (Kordes et al., 2013). Since it is claimed that ITGA5 preferably dimerizes with ITGB1 to form a functional integrin heterodimer (Nagae et al., 2012), *Itgb1* was also knocked out by CRISPR/Cas9 in freshly isolated HSC (gRNA 3) which were then exposed to shear stress (15 dyn/cm^2^). The knockout efficiency of gRNA 3 amounted to 98% (Figure 6h,i). HGF release by HSC with *Itgb1* knockout decreased significantly compared to mock transfection (Figure 6j). In addition, the basal level of HGF released under static condition (0 dyn/cm^2^) was significantly reduced in culture medium from HSC with *Itgb1* knockout in comparison to mock transfection. As seen for the *Itga5* knockout, the knockout of *Itgb1* also impaired cell adhesion significantly (Figure 6k). Reduced HGF release and impaired cellular adhesion due to knockout of *Itga5* and *Itgb1* indicate involvement of these integrins in mechanosensing of HSC.

## 3 DISCUSSION

In the present study, compelling evidence was obtained that the rat liver tissue is significantly altered during aging as demonstrated by decreased protein levels of collagens, laminins, elastin, fibronectin, HGF, and ITGA5. There were no signs of increased inflammation in the liver tissue and serum samples from old rats as determined by gene expression and protein arrays. Irrespective of this, the expression of inflammation‐associated factors was found to be elevated in HSC isolated from aged rat liver. Senescent cells secrete a specific pattern of chemokines, interleukins, and growth factors also known as SASP (Coppé et al., 2008; Freund, Orjalo, Desprez, & Campisi, 2010). These factors induce senescence and the release of SASP factors by neighboring cells in a paracrine manner (Acosta et al., 2013). HSC isolated from aged rats exhibited signs of SASP as indicated by elevated levels of CXCL1, CXCL3, and IL6. In contrast to these cytokines related to inflammation, the expression of growth factors such as *Hgf* decreased in HSC from old rats. This finding is in line with previous studies that reported decreased HGF levels in the aged liver (Enkhbold et al., 2015; Zhu et al., 2014). In contrast to this, fibroblasts of the skin have been described to reduce their cell size and to elevate HGF expression during aging (Qin, Worthen, & Quan, 2017), indicating that age‐related changes apparently differ among organs.

The reduced expression of quiescence‐associated markers such as *Gfap, Sparcl1*, and peroxisome proliferator‐activated receptor γ (*Pparγ*) in HSC from aged rats indicated impaired HSC quiescence and function. However, their phenotype was different from fibrosis‐associated myofibroblast‐like cells, since the expression of *α‐Sma*, desmin, and ECM‐associated genes remained unaffected or even declined in HSC during aging. In line with the loss of quiescence, HSC from aged rat liver indicated increased migration and impaired adhesion, since they upregulated the expression of cell migration‐associated factors such as the chemokine receptor *Cxcr4* and *Mmp13* whereas the focal adhesion elements *Vcl* and *Tln* as well as diverse integrin subunits were downregulated. CXCR4 is upregulated in activating HSC and enables a CXCL12 (stromal cell‐derived factor‐1)‐mediated cell migration (Sawitza et al., 2009). Increased HSC migration in aged liver was further supported by GO term analysis when differentially expressed genes in HSC from young and old rats were analyzed regarding their biological functions. However, this migration process is apparently not associated with an induction of ECM synthesis and loss of lipid‐containing droplets.

HSC fulfill a dual role in the regenerating liver. They release multiple factors including HGF to stimulate the proliferation of neighboring cells such as hepatocytes and they can differentiate into hepatocytes and cholangiocytes as demonstrated by transplantation and cell lineage‐tracing studies in rodents with injured liver (Kordes, Sawitza, Götze, Herebian, & Häussinger, 2014; Kordes et al., 2015; Michelotti et al., 2013; Swiderska‐Syn et al., 2014). Their ultimate behavior is most likely controlled by environmental factors and, thus, by their stem cell niche. The importance of the presence of specific ECM proteins in the microenvironment for the maintenance of HSC characteristics was demonstrated recently, since it has been shown that LN‐521 promotes the quiescent state of isolated HSC and that the LAMA5 chain is present in the space of Disse (Rohn et al., 2018). Although the LAMA5 amount remained unchanged during aging as observed in the present study, LAMB2 and LAMC1 showed a marked reduction in the liver tissue of old rats. These two laminin chains together with LAMA5 constitute the functional LN‐521 trimer. The present study provides evidence that *Hgf* gene expression and HGF protein release is supported by the culture of HSC on LN‐521. Furthermore, it has been shown previously that HGF is bound to the hepatic ECM as pro‐HGF (Masumoto & Yamamoto, 1991). Tissue section analyses of livers from young and old rats demonstrated the presence of HGF in liver sinusoids and indicated reduced HGF levels in the aged liver. In line with this, Western blot analyses of liver tissue from 2‐month‐old and 22‐month‐old rats revealed less pro‐HGF in the aged liver. This finding can be explained by the reduced ECM protein amount in combination with attenuated *Hgf* expression in HSC during aging, resulting in a reduced HGF pool available for liver regeneration. These results could explain the lowered regenerative potential of the aged liver after PHx that has been described by several groups for humans and rodents (Biondo‐Simões Mde et al., 2006; Enkhbold et al., 2015; Zhu et al., 2014).

The liver blood flow decreases by about 20%–55% during aging (Le Couteur & McLean, 1998) but increases shortly after liver injury by PHx (Lorenz et al., 2018). Plasma HGF concentration reaches a peak at about one hour and decreases during the first day after PHx while remaining elevated for more than three days (Lindroos et al., 1991). The rise of HGF plasma levels after liver injury is based on ECM‐bound pro‐HGF unleashed from the ECM by MMP (Kim et al., 1997). In the present study, analyses of rat liver tissue sections showed markedly reduced HGF levels one day after PHx compared to normal control liver. Recently, it was demonstrated that the immediate increase of HGF plasma levels involves also SEC that release HGF after exposure to mechanical forces exerted by increased blood flow after PHx (Lorenz et al., 2018). To analyze whether this mechanism also applies for perfused rat liver and isolated HSC, fluid mechanical forces were applied. After elevating the perfusion rate via the portal vein, HGF release from liver sinusoids was detected, similar to the situation after PHx. Furthermore, HGF release by isolated HSC was increased by applying shear stress and stretching. At present, the exact fluid shear forces within liver sinusoids and the space of Disse in vivo remain unknown. For liver sinusoids, 3.7 dyn/cm^2^ was calculated (Noh et al., 2012). Therefore, a wide range of shear forces ranging from 2.9 to 29 dyn/cm^2^ was used in this study, which all triggered the release of HGF by HSC. Sensing of mechanical stimuli can be transmitted by integrins to downstream signaling pathways (Häussinger, Reinehr, & Schliess, 2006). For example, the hepatocyte volume increases under the influence of insulin, which leads to an integrin‐dependent epidermal growth factor receptor and ERK1/2 activation in hepatocytes, triggering a proliferative response (Reinehr, Sommerfeld, & Häussinger, 2010; Schliess, Reinehr, & Häussinger, 2007). Adhesion of cells to the ECM protein FN is mediated via a specific binding of the ITGA5/ITGB1 heterodimer (Nagae et al., 2012). Interestingly, FN and ITGA5 were markedly downregulated in liver tissue and HSC from old rats indicating impaired cell‐ECM interactions. In contrast, when isolated HSC were exposed to fluid shear stress, several genes with decreased expression in aged rat liver (*Col1a2*, *Lamb1*, *Lamb2*, *Lamc1*, *Itga5*) were upregulated. Knockout of *Itga5* and *Itgb1* in HSC led to reduced cell adherence and HGF release. Furthermore, *Itga5* knockout abolished the increased expression of ECM‐associated genes after exposure to shear stress. These results suggest that ITGA5/ITGB1 is a mechanosensor in HSC. However, since the shear stress‐induced reduction of *Il6* expression in HSC persisted after *Itga5* knockout, additional mechanisms for sensing of mechanical stimuli in HSC can be assumed.

In conclusion, HGF and several components of the liver matrix, such as collagens, laminins, and FN which maintain HSC characteristics and enhance their HGF expression, were significantly reduced in the aged liver as shown in this study. In concert with this, HSC from old rats showed downregulated HGF expression and signs of senescence. Interestingly, the expression of laminins and collagens as well as the release of HGF by HSC were found to depend on mechanical forces. Mean *Itga5* and *Itgb1* mRNA amounts were markedly reduced in HSC from aged rat liver. Knockout of *Itga5* and *Itgb1* in HSC impaired cell adhesion and reduced HGF release. *Itga5* knockout additionally prevented elevated expression of laminins and collagens after exposure to shear stress. Since other integrins and the adherence of HSC decrease during aging, impaired mechanosensing and signal transduction in HSC may not only depend on ITGA5/ITGB1. Nevertheless, the present study provides an interesting link between shear stress, HSC function, and age‐related changes of liver homeostasis, demonstrating that physical factors belong to the niche elements of HSC in the space of Disse. Reduced fluid mechanical forces together with an altered ECM composition in the space of Disse and a reduction of ITGA5/ITGB1 in HSC seem to affect proper HSC function such as HGF release which might contribute to the impaired regenerative potential of the aged liver.

## 4 EXPERIMENTAL PROCEDURES

### 4.1 Liver samples, cell isolation, and culture

Liver tissue and HSC isolation were derived from 2‐month‐old (343 ± 5 g) and 22‐month‐old (598 ± 19 g) male Wistar rats from Janvier Labs (France). Initially, the blood was harvested from the portal vein for further analyses. Samples from three different lobes (median lobe, left lateral lobe, and right lateral lobe) of each liver were collected and pooled for further analysis. To isolate HSC, the livers were perfused with collagenase and pronase E solutions, in order to digest the liver tissue essentially as described (Hendriks, Verhoofstad, Brouwer, Leeuw, & Knook, 1985). After enrichment of HSC by density gradient centrifugation using 28% Nycodenz (Nycomed Pharma, Oslo, Norway), the HSC were purified by fluorescence‐activated cell sorting (FACS) using their typical retinoid fluorescence emitted after UV‐light excitation (FACS Aria III, BD Biosciences, Heidelberg, Germany).

The sorted HSC were cultured for 24 hr prior to collection for RNA isolation in Iscove's Modified Dulbecco's Medium (IMDM; Thermo Fisher Scientific) supplemented with 10% FCS and 1% antibiotic–antimycotic solution (Thermo Fisher Scientific). The HSC isolation protocol was authorized by the Landesamt für Natur, Umwelt und Verbraucherschutz (Recklinghausen; 82‐02.04.2015.A287).

For in vitro experiments, HSC were isolated from mid‐aged Wistar rats (> 500 g; 10–15 months), which were obtained from the animal facility of the Heinrich Heine University Düsseldorf (Germany). HSC were first cultured on plastic culture dishes in Dulbecco's Modified Eagle's Medium (DMEM) supplemented with 10% FCS and 1% antibiotic–antimycotic solution. After one day, HSC were detached by trypsin/EDTA solution and finally seeded into µ‐Slides I^0.4^ precoated with COL4 (ibidi, Martinsried, Germany; 250,000 cells in 100 µl medium). Alternatively, 800,000 HSC were seeded on silicone stretch chambers (STREX) precoated with 1.0 µg/cm^2^ COL4 (Sigma‐Aldrich). After 24 hr, medium was exchanged to serum‐free IMDM supplemented with 1% insulin‐selenite‐transferrin (ITS; Sigma‐Aldrich) containing albumin and linoleic acid as well as 1% antibiotic–antimycotic solution.

### 4.2 Culture of HSC on protein‐coated surfaces

HSC were seeded (50,000 cells/cm^2^) either on uncoated polystyrene culture dishes or on dishes coated (1.0 µg/cm^2^) with fibronectin (FN, Sigma‐Aldrich), COL4 (Sigma‐Aldrich), laminin‐211 (LN‐211), or laminin‐521 (LN‐521) obtained from BioLamina. After 24 hr, culture medium was changed to the serum‐free medium. After seven days of culture, cells and culture media were collected for further analyses. In a different approach, nanostructured surfaces were generated by binding laminin to gold dots with an average spacing of 151 ± 35 nm. These gold dots were bio‐functionalized with LN‐211 or LN‐521 (BioLamina) as described (Rohn et al., 2018). HSC from mid‐aged rats were seeded directly after isolation on nanostructured surfaces and cultured under serum‐free conditions. After one week, HSC were harvested for qPCR analyses.

### 4.3 CRISPR/Cas9‐mediated knockout of *Itga5* and *Itgb1*

Guide RNA (gRNA) for *Itga5* and *Itgb1* were designed using the program Chopchop V2 (Labun, Montague, Gagnon, Thyme, & Valen, 2016) and synthesized with the GeneArt Precision gRNA Synthesis Kit (Invitrogen). The target sequences of the *Itga5* and *Itgb1* genes are listed in Table S7. The transfection of HSC from mid‐aged rats was performed with the TrueCut Cas9 Protein v2 (Invitrogen) together with the gRNA using the P3 Primary Cell 4D‐Nucleofector X Kit L (Lonza, Basel, Switzerland) and the 4D‐Nucleofector (Lonza; program DS167). As a control (mock), HSC were transfected in the presence of Cas9 protein but gRNA was absent. Subsequently, HSC were seeded on standard culture dishes and harvested after 3 days to determine the knockout efficiency by Western blot. For shear stress experiments (15 dyn/cm^2^), HSC with and without CRISPR/Cas9‐mediated *Itga5* and *Itgb1* knockout were seeded in µ‐Slides I^0.4^ precoated with COL4 (ibidi) as described above.

### 4.4 Gene expression analyses by microarray and qPCR

Total RNA of HSC and whole liver tissue from three young (2 months) and three old (22 months) rats was extracted using the RNeasy Mini Kit according to manufacturer's instructions (Qiagen) and sent to IMGM Laboratories for gene expression analyses by microarrays (Affymetrix, GeneChip Rat Gene 2.0 ST Array, Thermo Fisher Scientific). The array data were processed by the Transcriptome Analysis Console 3.0 (Thermo Fisher Scientific) and are available online at ArrayExpress database (http://www.ebi.ac.uk/arrayexpress) under the accession number: E‐MTAB‐7423. Gene ontology (GO) term analysis of significantly altered genes with regard to biological processes was performed with the GO enrichment analysis and visualization software GOrilla (October 2018; http://cbl-gorilla.cs.technion.ac.il/; Eden, Navon, Steinfeld, Lipson, & Yakhini, 2009). Furthermore, RNA from liver tissue of additional rats was isolated and the Revert Aid H Minus First Strand cDNA Synthesis Kit (Thermo Fisher Scientific) was used for cDNA synthesis according to manufacturer's instructions. The qTower3 cycler (Analytik Jena AG) and the Maxima SYBR Green qPCR Mastermix (Thermo Fisher Scientific) were used for qPCR analyses. The sequences of all expression primers are listed in Table S8. The qPCR raw data were normalized to the Ct values of the housekeeping gene hypoxanthine‐guanine phosphoribosyl transferase 1 (*Hprt1*) and the ΔΔ*Ct*‐method was used for the calculation of relative expression changes.

### 4.5 Immunofluorescence analysis

Cryosections of young, old, regenerating (PHx), and perfused rat livers as well as isolated HSC under static and shear stress conditions were fixed in ice‐cold methanol or 4% formalin (*n* = 3). The following primary antibodies were used: COL1 (C2456; Sigma‐Aldrich), COL4 (ab6586; Abcam), LAMA5 (NBP2‐42391; Novus Biologicals), FN (610078; Becton Dickinson), ITGA5 (ab150361; Abcam), ITGB1 (ab52971; Abcam), HGF (80429‐R052; Sino Biological), GFAP (MAB3402; Merck/Millipore), desmin (ab8592; Abcam), and α‐SMA (M0851; Dako/Agilent). The samples were then incubated with anti‐mouse or anti‐rabbit secondary antibodies labeled with Cy3 or FITC (Millipore). The tissue sections were mounted with Fluoromount G containing 4′,6‐diamidino‐2‐phenylindole (DAPI; Southern Biotech) and a glass coverslip. Images were taken using an Olympus IX50 microscope equipped with a DP71 camera (Olympus). The same exposure time was used for each antibody to enable comparison of liver sections from different age groups.

### 4.6 Western blot analysis

Protein analyses of whole liver tissue lysates were performed using the semidry Western blot technique according to standard protocols. Antibodies against α‐SMA (M0851; Dako/Agilent), desmin (ab8592; Abcam), GFAP (MAB360; Merck/Millipore), fibronectin (610078; Becton Dickinson), ITGA5 (ab150361; Abcam), ITGB1 (ab52971; Abcam), HGF (LS‐C486534; Lifespan Biosciences), and γ‐tubulin (T5326; Sigma‐Aldrich) were used. The detection was carried out using horseradish peroxidase (HRP)‐coupled goat‐anti‐mouse or goat‐anti‐rabbit antibodies (Merck/Millipore) and Western Bright Quantum or Sirius HRP substrates (Advansta). Visualization and documentation was performed with ChemiDoc Imaging System (BioRad). The intensities of Western blot protein bands were analyzed using the multiplex band analysis tool of the AlphaView software (Proteinsimple) and normalized to the intensity of γ‐tubulin bands.

### 4.7 Mass spectrometry‐based label‐free quantification of ECM

The liver was perfused with PBS without Ca^2+^/Mg^2+^ for 20 min and then three times with 500 ml PBS with Ca^2+^/Mg^2+^ containing increasing concentrations of Triton X‐100 (1%, 2%, and 3%; Sigma‐Aldrich). Subsequently, perfusion was performed three times with 1,000 ml PBS with Ca^2+^/Mg^2+^ supplemented with increasing concentrations of sodium dodecyl sulfate (SDS; 0.1%, 0.5%, and 1%) each containing 20 mg DNase (Sigma‐Aldrich). Finally, decellularized liver tissue was flushed with deionized water. From each rat liver, ECM pieces from three different liver lobes were pooled and lyophilized for bottom‐up mass spectrometric analysis essentially as described for muscle cell cultures (Grube et al., 2018). Briefly, tissue lysates were prepared and 5 µg per sample shortly (~5 mm running distance) stacked in a polyacrylamide gel. After staining and band processing, proteins were digested with trypsin and peptides extracted from the gel and finally prepared for mass spectrometry. Here, peptides were separated by an Ultimate 3000 rapid separation liquid chromatography system by a two‐hour gradient and injected into a QExactive plus mass spectrometer via a nano‐electrospray interface and measured with a top 10 method essentially as described (Grube et al., 2018). Data analysis was carried out within the MaxQuant environment (version 1.6.1.0; MPI for Biochemistry) with standard parameters. Searches were carried out using the *Rattus norvegicus* proteome set (UP000002494, 29,966 entries) from the Uniprot Knowledgebase downloaded on April 18, 2018. The matched between runs function was enabled as well as label‐free quantification. Only proteins showing at least 2 different peptides and three valid values in at least one group were selected for further analysis of quantitative data.

### 4.8 Analysis of SASP factors in serum and HSC culture medium

For the detection of SASP factors in the serum (*n* = 4) or serum‐free conditioned culture medium of isolated HSC (*n* = 3) from young and old rats, the Proteome Profiler Rat Cytokine Array Kit (R&D Systems) was used according to the manufacturer's instructions. Analysis of serum samples revealed that 10 out of 29 factors showed a reaction on the membrane while only two factors could be detected on the membrane incubated with the culture medium. Visualization and documentation were performed with ChemiDoc Imaging System (BioRad) and protein dot intensities were analyzed with the AlphaView software (Proteinsimple). Furthermore, culture supernatants of HSC from young (*n* = 5) and old (*n* = 3) rats were analyzed with the Rat CXCL3/CINC‐2 alpha/beta Quantikine ELISA Kit (RCN200, R&D Systems) and the Rat IL6 Quantikine ELISA Kit (R6000B, R&D Systems) according to manufacturer's recommendations. Data from the cytokine array and ELISA analyses were normalized to cell number per area, which was assessed by counting the cells with the cellSens Dimension 1.16 software at three different positions of each culture dish (Olympus). To investigate the amount of HGF released by HSC after shear stress (*n* = 5) and stretching (*n* = 4) as well as by culture on different ECM proteins, culture supernatants were analyzed with the Mouse/Rat HGF Quantikine ELISA (R&D Systems).

### 4.9 Partial hepatectomy

After anesthesia of male Wistar rats (approximately 250 g body weight, 8–10 weeks), PHx was performed by surgical removal of the two largest liver lobes (70% of the liver mass) as described (Higgins & Anderson, 1931; Kordes et al., 2014). Tissue samples were taken after liver perfusion with physiologic buffer one day after PHx to analyze HGF within the regenerating tissue by immunofluorescence. HGF fluorescence intensity was quantified with the cellSens Dimension 1.16 software from six different positions of each liver section (Olympus). Liver tissues from three control and PHx rats were analyzed. Permission for the animal experiments was given by the Landesamt für Natur, Umwelt und Verbraucherschutz (9.93.2.10.34.07.163).

### 4.10 Liver perfusion

Rat livers from young and old male Wistar rats were perfused via the portal vein using Krebs–Henseleit buffer essentially as described (Wettstein & Häussinger, 1997). The flow rate of the buffer was adjusted to the liver weight. Initially the liver was perfused with 2 ml/(min × g liver) and after 10 min the perfusion rate was increased to 6 ml/(min × g liver). The liquid pressure was continuously recorded during liver perfusion. The effluent was collected in fractions to enable quantification of HGF by the Mouse/Rat HGF Quantikine ELISA (R&D Systems). The liver perfusion experiments were approved by the Landesamt für Natur, Umwelt und Verbraucherschutz (81‐02.04.2018.A126).

### 4.11 Mechanical stimulation of isolated HSC

Freshly isolated HSC at day 3 of culture were exposed to shear stress (2.9, 15, or 29 dyn/cm^2^) for 1 hr under laminar, pulsatile flow using the “yellow/green” perfusion set (ibidi). For mimicking the heartbeat of rats, a pulsatile flow at 2.5 Hz was used which represented the maximum pulse rate of the ibidi perfusion system. The media from µ‐Slides under static condition (0 dyn/cm^2^) were collected as a reference. HSC within the flow chamber were lysed for RNA extraction or fixed for immunofluorescence analysis. For cell stretching, the culture supernatant was aspirated and 1 ml of fresh serum‐free medium was added. Stretch chambers were either elongated by about 30% or remained unstretched as a control for 1 hr. All culture supernatants were collected from static and shear stress experiments as well as from unstretched and stretched HSC.

### 4.12 Statistics

Values from 3–13 different animals per age group are presented as means and their variation is indicated by the standard error of the mean (±*SEM*). Significant differences between age groups were determined by nonparametric tests (Mann–Whitney test *U* test) unless otherwise stated. Only *p* values smaller than .05 were considered significant. To distinguish significantly different groups, either an asterisk (*) or letters (a, b, c, d) were used. If groups share the same letters, no significant differences were found. Principal component analysis (PCA) on microarray raw data was conducted with the software Transcriptome Analysis Console 4.0.0.25 (AppliedBiosystems, Thermo Fisher Scientific).

## CONFLICT OF INTEREST

The authors declare no conflict of interest.

## AUTHOR CONTRIBUTIONS

FR performed experiments, analyzed data, and wrote the manuscript; CK designed study, performed experiments, analyzed data, and wrote the manuscript; TB performed stretch experiments and analyzed data; DR performed experiments and analyzed data; MW performed FACS and analyzed data; GP and KS performed proteome analyses and approved the manuscript; AB, FG, and JS generated laminin‐functionalized nanostructured surfaces and approved the manuscript; and DH designed study, analyzed data, and wrote the manuscript.

## Supporting information

Supplementary Material

## Data Availability

The authors provide detailed description of methods and original data upon request.
