# Supplementary material for: Impaired integrin α5/β1‐mediated hepatocyte growth factor release by stellate cells of the aged liver
Source: Aging Cell. 2020 Mar 11;19(4):e13131. doi: 10.1111/acel.13131 (PMC7189994; doi:10.1111/acel.13131)
Supplement: Supplementary file 1 — Supplementary Material [file ACEL-19-e13131-s001.pdf]

Impaired integrin α_5/β_1-mediated hepatocyte growth factor release by stellate cells of the aged liver

Friederike Rohn[1*], Claus Kordes[1*], Tobias Buschmann[1], Doreen Reichert[1], Marianne Wammers[1], Gereon Poschmann[2], Kai Stühler[2, 3], Amelie S. Benk[4,5], Fania Geiger[4,5], Joachim P. Spatz[4,5], Dieter Häussinger[1]

*These authors contributed equally.

[1]Clinic of Gastroenterology, Hepatology and Infectious Diseases,
Heinrich Heine University, Düsseldorf, Germany

[2]Institute for Molecular Medicine, Heinrich Heine University, Düsseldorf, Germany

[3]Molecular Proteomics Laboratory, BMFZ, Heinrich Heine University Düsseldorf, Düsseldorf, Germany

[4]Max-Planck-Institute for Medical Research, Department of Cellular Biophysics, Jahnstraße 29, 69120 Heidelberg, Germany

[5]Department of Biophysical Chemistry, University of Heidelberg, Im Neuenheimer Feld 253, 69120 Heidelberg, Germany

Supplemental data

Supplemental Table S1: Differential expression of markers associated with ECM in whole liver tissue from aging rats. The gene expression in liver tissue from young (2 months) and old (22 months) rat livers was investigated by Affymetrix micorarrays (n = 3 for each group). Negative fold change implicates lower expression in hepatic tissue from old rats ($p < 0.05$). All ECM-associated genes, that were significantly altered in the liver tissue from 22-months-old rats, showed a reduction. Among integrins, only integrin α_5 (*Itga5*) was markedly regulated.

Process	Gene Symbol	Fold Change	ANOVA p-value
ECM-associated	Lama2	-1.31	0.026214
	Nid1	-1.82	0.000481
	Col1a1	-1.23	0.002631
	Col1a2	-2.92	0.014069
	Col3a1	-2.50	0.001962
	Col4a1	-1.54	0.011863
	Col5a1	-1.41	0.028843
	Col14a1	-1.20	0.010225
	Eln	-1.55	0.022032
	Fn1	-1.05	0.047903
integrins	Itga1	-1.09	0.026565
	Itga5	-1.65	0.002914
	Itga6	-1.37	0.049957
	Itgb4	1.17	0.044354

Supplemental Table S2: Matrisome analysis of decellularized rat liver.
(see separate Excel file “Supplemental Table S2”)
Two separate tables are given: one table containing all identified proteins and one table containing all quantified proteins. The tables include data from protein identification as was as quantitative data from label-free quantification.

Supplemental Table S3: Differential expression of markers associated with ECM and integrins in HSC from aging rats. The gene expression of HSC isolated from young (2 months) and old (22 months) rat livers was investigated by Affymetrix micorarrays (n = 3 for each group). Negative fold change implicates lower expression while positive values indicate higher expression in HSC from old rats ($p < 0.05$). The expression of *Mmp13* increased markedly on mRNA level in array analysis, whereas matrix proteins such as *Lama2*, *Lamc1*, *Nid1*, *Nid2*, collagens, and *Fn1* decreased in HSC from old rats. Furthermore, several integrins were significantly downregulated such as *Itga5*.

Process	Gene Symbol	Fold Change	ANOVA p-value
ECM-associated	Mmp2	-2.68	0.004478
	Mmp28	-1.67	0.004404
	Mmp17	1.21	0.029466
	Mmp16	1.24	0.025820
	Mmp3	1.74	0.000433
	Mmp11	2.37	0.001967
	Mmp13	15.38	0.000619
	Lama2	-3.15	0.000760
	Lamc1	-1.38	0.006252
	Lama4	2.92	0.009783
	Nid2	-3.37	0.002714
	Nid1	-1.58	0.000652
	Col8a1	-9.65	0.002631
	Col12a1	-7.79	0.000108
	Col1a2	-7.47	0.000010
	Col3a1	-4.35	0.000293
	Col1a1	-3.90	0.000268
	Col5a2	-3.23	0.000345
	Col4a1	-2.39	0.000551
	Col4a2	-2.14	0.000920
	Col5a1	-2.12	0.000001
	Col14a1	-1.95	0.017937
	Col6a3	-1.88	0.021628
	Col6a2	-1.56	0.007514
	Fn1	-7.89	0.000032
integrins	Itga2	-2.54	0.005192
	Itga9	-2.12	0.027800
	Itga5	-1.96	0.000357
	Itga6	-1.72	0.027102
	Itga3	-1.50	0.010345
	Itgb1	-1.48	0.000374
	Itga1	-1.29	0.004938
	Itga4	-1.24	0.024648
	Itgax	2.18	0.011056
	Itgb2	2.34	0.003577
	Itgb8	4.88	0.001907

Supplemental Table S4: Gene ontology (GO) term analysis with regard to biological processes of significantly up or downregulated genes in HSC obtained by microarray. (see separate Excel file “Supplemental Table S5”)

Supplemental Table S5: Differential expression of markers associated with quiescence or activation in HSC from aging rats. The gene expression of HSC isolated from young (2 months) and old (22 months) rat livers was investigated by Affymetrix microarrays (n = 3 for each group). Negative fold change implicates lower expression while positive values indicate higher expression in HSC from old rats. The quiescent-associated markers Sparcl1, Pparγ, and reelin as well as activation-associated markers fibronectin, periostin, nestin, and desmin were significantly downregulated in HSC from old rats ($p < 0.05$), while Gfap and α-Sma remained unchanged on mRNA level in array analysis.

Process	Gene Symbol	Fold Change	ANOVA p-value
HSC quiescence / activation-associated	fibronectin	-7.89	0.000032
	nestin	-2.96	0.008251
	Sparcl1	-2.84	0.000004
	desmin	-2.22	0.002086
	periostin	-2.18	0.002297
	Pparγ	-1.60	0.024991
	reelin	-1.47	0.007415
	α-Sma (Acta2)	1.04	0.666481
	Gfap	1.20	0.098913

Supplemental Table S6: Differential expression of markers associated with SASP in HSC from aging rats. The gene expression of HSC isolated from young (2 months) and old (22 months) rat livers was investigated by Affymetrix microarrays (n = 3 for each group). Negative fold change implicates lower expression while positive values indicate higher expression in HSC from old rats ($p < 0.05$). Many genes associated with SASP exhibited altered expression in HSC from old rats, when compared to HSC from young rats. The expression of many growth factors declined, but inflammation- and cell migration-associated genes exhibited elevated expression in array analysis.

Process	Gene Symbol	Fold Change	ANOVA p-value
Senescence-associated secretory phenotype (SASP)	Tgfb3	-4.87	0.00547
	Tgfb2	-3.02	0.00121
	Timp2	-2.70	0.00015
	Tgfb1	-2.16	0.00033
	Fgf2	-1.75	0.00054
	Ctgf	-1.72	0.00012
	Hgf	-1.68	0.03020
	Il15	-1.64	0.02986
	Igfbp7	-1.10	0.00747
	Adipoq	-1.10	0.03161
	Ccl26	-1.06	0.04205
	Cxcl3	1.33	0.00091
	Il6st	1.46	0.00021
	Icam1	1.49	0.00853
	Ccl2	1.50	0.02335
	Cxcl12	1.56	0.02179
	Ctsb	1.56	0.00038
	Icam2	1.64	0.01713
	Tnfrsf1a	1.65	0.00060
	Tnfrsf1b	1.76	0.00376
	Ngf	1.91	0.00096
	Cxcl10	1.93	0.00122
	Cxcl9	1.93	0.02212
	Ereg	2.09	0.00989
	Cxcl5	2.28	0.00099
	Cxcl1	3.70	0.00050
	Il6	4.01	0.01542
	Ccl20	4.57	0.00833
	Cxcl13	7.75	0.00124
	Serpinb2	7.90	0.05005
	Csf2	8.87	0.00458
	Cxcr4	10.15	0.00076
	Cxcl2	10.16	0.00308
	Il1a	14.13	0.00098
	Csf3	23.86	0.00169

Supplemental Table S7: CRISPR/Cas9 target sequences for *Itga5* and *Itgb1* genes.

Gene	gRNA no.	Target sequence
Itga5	gRNA 1	GGGCTTCAACCTAGACGCGGAGG
	gRNA 2	CTCCGTGGAGTTTTACCGGCCGG
Itgb1	gRNA 3	GTTGGTCAGCAGCGCATATCTGG

Supplemental Table S8: Primer sets for qPCR analysis.

Gene	Accession no.	Forward primer	Reverse primer
α-Sma	NM_031004	GCACTACCATGTACCCAGGCA	TGCGTTCTGGAGGAGCAATA
Col1a2	NM_053356	ACCTCAGGGTGTTCAAGGTG	GGGATTCCAATAGGACCAGA
Col4a1	NM_001135009	GTCCTCACTGTGGATTGGCTA	AGTAATTGCACGTTCCTCTGC
Col6a6 *	XM_017596190	TTGCCCCAAACATGACACAG	TCTAGGTTCTCTTGGCTGCC
desmin	NM_02253	ACCTTCCGATCCAGACCTTCT	TTCATGTTGTTGCTGTGTGGC
Eln	NM_012722	GTGGCTATGGACTGCCCTATAC	CCTCCAGCAGCTCCATACTTAG
Fn	NM_019143	ATGTTGGAGTGTTTGTGTCTGG	GTGTCCTGATCATTGCATCTGT
Gfap	NM_017009	ACATCGAGATCGCCACCTAC	TCCACCGTCTTTACCACGAT
Hgf	NM_017017	CGAGCTATCGCGGTAAAGAC	TGTAGCTTTCACCGTTGCAG
Hprt1	NM_012583	AAGTGTTGGATACAGGCCAGA	GGCTTTGTACTTGGCTTTTCC
Il6	NM_012589	TACCCCAACTTCCAATGCTC	GGTTTGCCGAGTAGACCTCA
Itga1	NM_030994	CGAGCCTCTGGCTTCTTATTT	GATGTTCCCATCCTCCATCTT
Itga2	XM_001075558	CCACAGAAGCTTCGTTCACTC	GGTCAGCATCAAGTGTCATGTT
Itga3	XM_006247215	CCCTCAAGAACGATTGTGAAC	AGACCACAGCACCTTGGTGTA
Itga5	NM_001108118	CCCAAAGGAAACCTCACCTAT	TCATCTAGCCCATCTCCATTG
Itga6	NM_053725	GGCTCTTGTCAGCAAGGAGTA	CAACTTCATAGGGCCCATCTT
Itga11	NM_001108156	ACAGCATCGAGTGTGTGAATG	ATGGATCTGAAGATGGTGCAG
Itgb1	NM_017022	TTGGACACTGTTCCATGCGTA	TTGAAGGCTCTGCACTGAACA
Itgb3	NM_153720	GGCCTCAAGATTGGAGACAC	GCAGTCACAGTCACAGTCGAA
Itgb4	NM_013180	CTGCTCATCTTCCTCCTGTTG	TCCCGCAGCATATAGTGATCT
Lama2	XM_017590488	GGGACACGAACGATGAGGAAA	TTTTCACTTCGATGGGCTGCT
Lama4	NM_001309447	ACTATGTCAGTGAGGCCAACG	CTGGCTTGATTGAGAAGGTTG
Lama5	NM_001191609	CAAATGAGCATCGCCTTCCTG	CCAGCACCATCATGAGTTCCT
Lamb1	XM_003750137	CGTGGACTCTGTGGAGAAGAA	CTCCGTGAAGCTGTGTCAGAT
Lamb2	NM_012974	CAGAGCTGGAATTGGTGGTG	TGTAGGAGAGACCAGGCTCAA
Lamc1	NM_053966	GCCTTTTCAACTCTGGAAGGA	ACTTGAGGACTTTGGGGTCAT
Lrat	NM_022280	GACCTACTGCAGATACGGCTC	TATGATGCCAGGCCTGTGTAG
Lu/Bcam	NM_031752	GAGGATTACGATGCTGACGAG	ATCTGCAACGGTCACAGAATC
nestin	NM_001308239	GATCGCTCAGATCCTGGAAG	AGGTGTCTGCAACCGAGAGT
Nid1	XM_213954	TGTGCCAACAATAGACACCAG	AAGATCCTTCCCTTCACCTTG
Nid2	NM_001012005	ATTCACCATGGAGGCAGTTC	CCGGGGGTATTGTAACAGATG
reelin	NM_080394	CTGCAATACAGCGTCAACAAC	CCACTGATCATGACCTGTTCC
Sparcl1	NM_012946	CCTCAAATACGGAGAGGAGAC	GTCCCCTTTTACACTGGAAGT

* The primer set detects the transcript variants 1-8 of *Col6a6*.

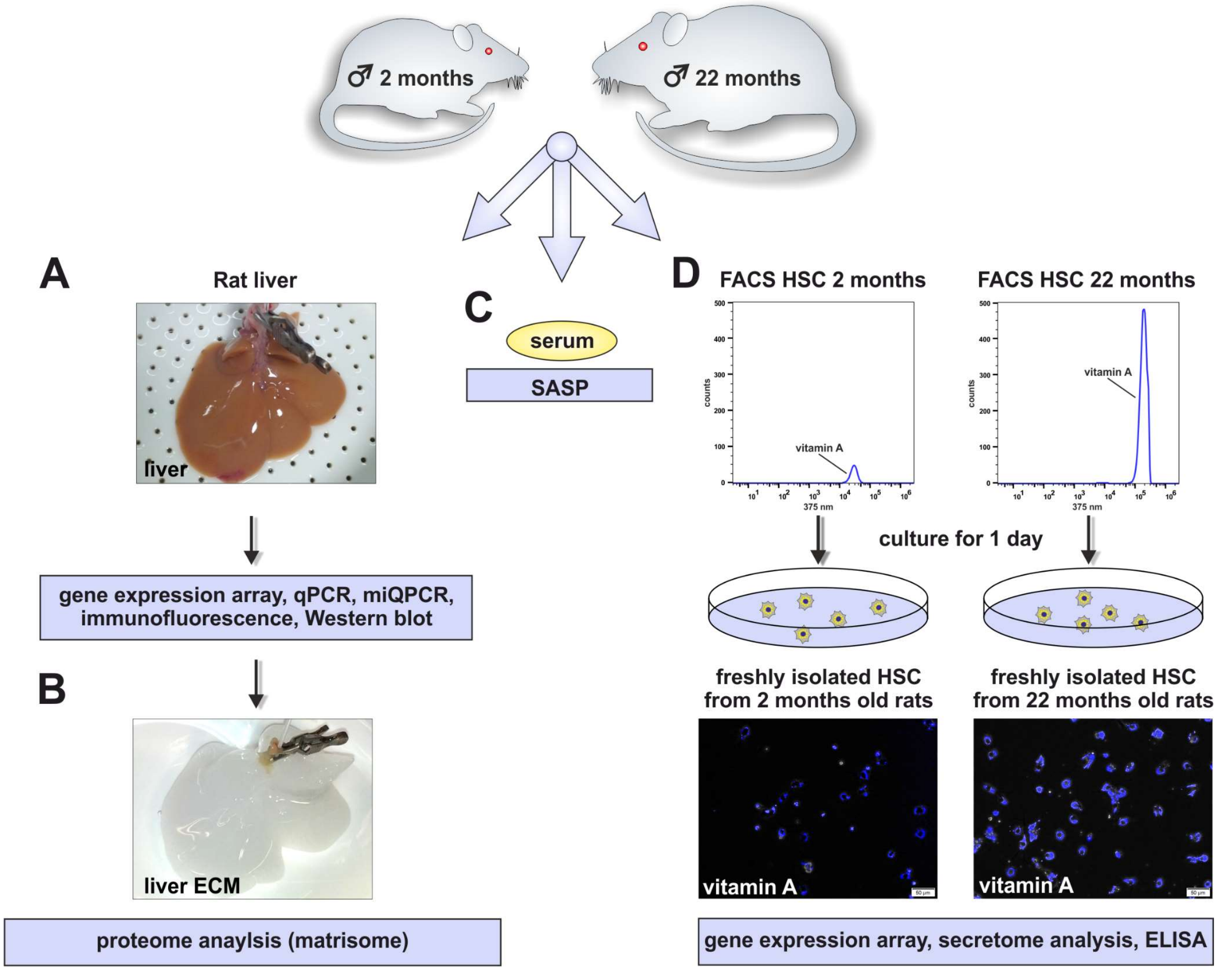

Supplemental Figure S1: Experimental setup. Isolated HSC and liver tissue from young (2 months) and old (22 months) rats were compared to unravel possible alterations of stellate cells and their niche during aging. **(A)** The liver tissues of young and old rats were also analyzed by microarrays (see above; n = 3 for each age group). The array results were evaluated by qPCR analysis and completed by immunofluorescence as well as Western blot to identify age-related alterations in the liver (n = 3-10). **(B)** To enable quantitative assessment of the matrisome, liver tissues from both age groups were decellularized for proteome analysis (n = 3 for both age groups). **(C)** The blood serum was collected from young and old rats and analyzed regarding cytokines indicating a SASP. **(D)** HSC of both age groups were isolated and enriched by density gradient centrifugation and further purified by FACS using their characteristic vitamin A (retinoid) fluorescence. After one day of culture, the HSC showed typical cell morphology and vitamin A fluorescence (blue). The HSC from young rats contained fewer retinoids compared to those obtained from old rats. The RNA of HSC was harvested and analyzed by microarrays (Affymetrix GeneChip Rat Gene 2.0 ST Array; n = 3 for each age group). The medium of the HSC was collected for protein arrays and ELISA to investigate their secretome with respect to a SASP.

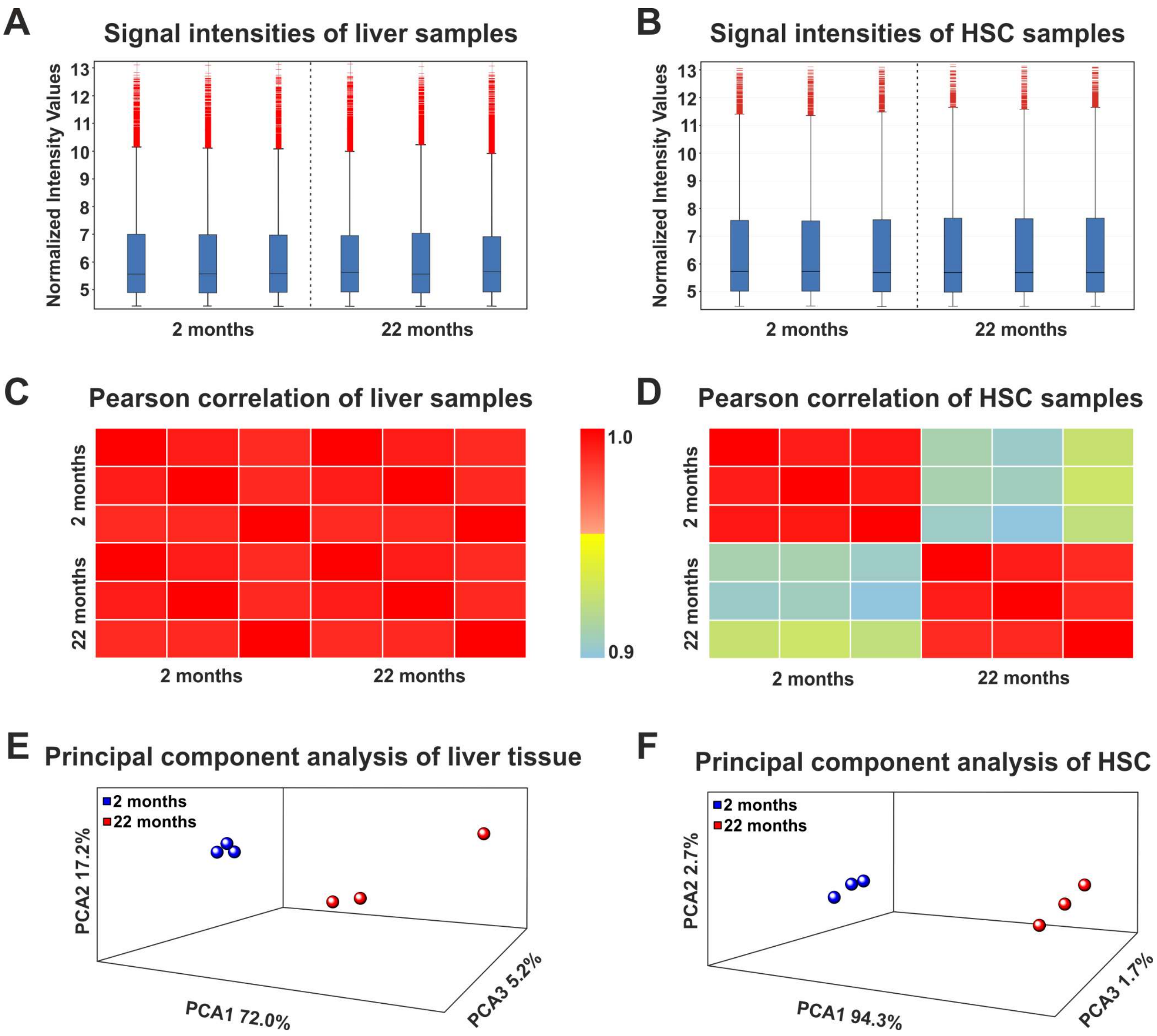

Supplemental Figure S2: Quality control of microarray raw data. (A, B) To evaluate the reliability of the data sets obtained by microarray analysis for liver tissue and isolated HSC (cultured for 1 day) from young (2 months) and old (22 months) rats, the signal intensities of samples on genes chips were compared. **(C, D)** Pearson correlation analysis of the microarray data for liver tissue and isolated HSC from both age groups was performed and revealed that the gene expression of liver samples seemed to be equal. In contrast, significant differences between HSC from young and old rats were observed when Pearson correlation was applied. **(E)** Gene expression data of whole liver tissue from both groups were compared by principal component analysis (PCA) and showed that the samples of young rats were highly similar and different from old rats. **(F)** PCA of gene expression arrays in isolated HSC from 2 (blue) and 22 (red) months old rats. HSC from young rats exhibited highly similar gene expression and were different from samples obtained from old rats, which clustered together, but exhibited a higher variation. Each dot represents a data set from a single animal (n = 3 for each age group).

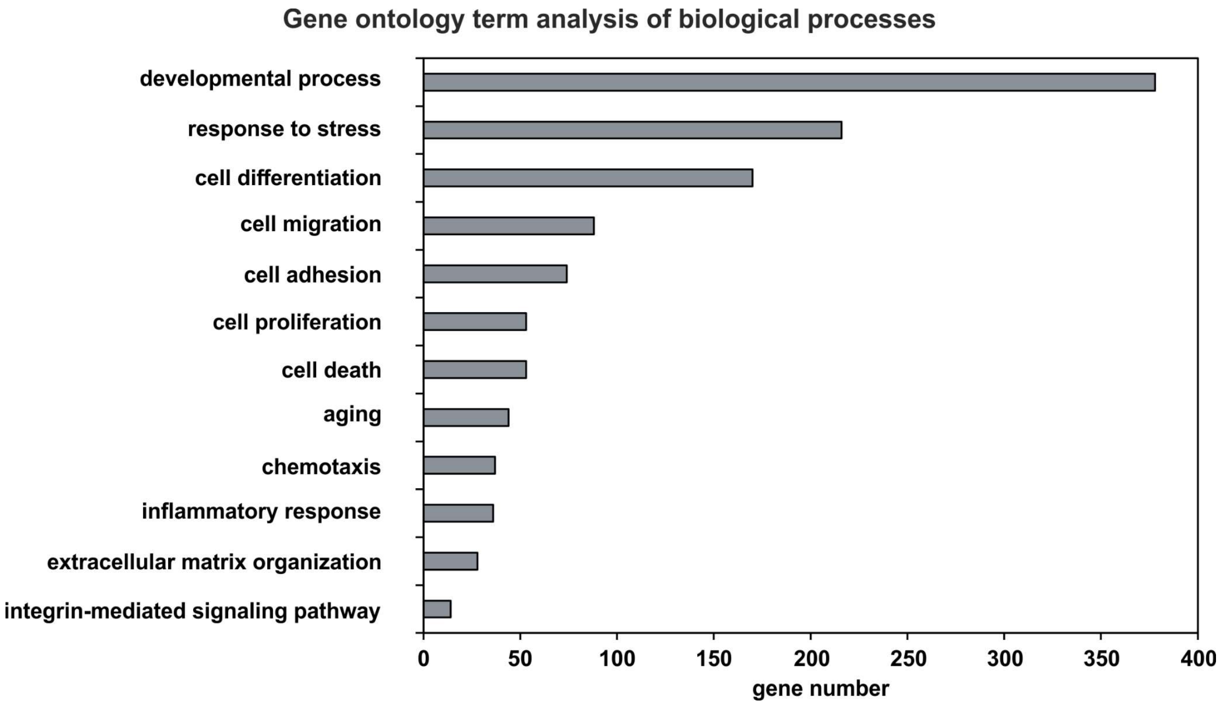

Supplemental Figure S3: Gene ontology (GO) term analysis of differentially expressed genes in HSC from old compared to young rats with respect to biological processes. GO term analysis of differentially expressed genes obtained by Affymetrix microarrays was performed with the software GOrilla (October 2018; fold enrichment > 1.5; $p < 0.05$).

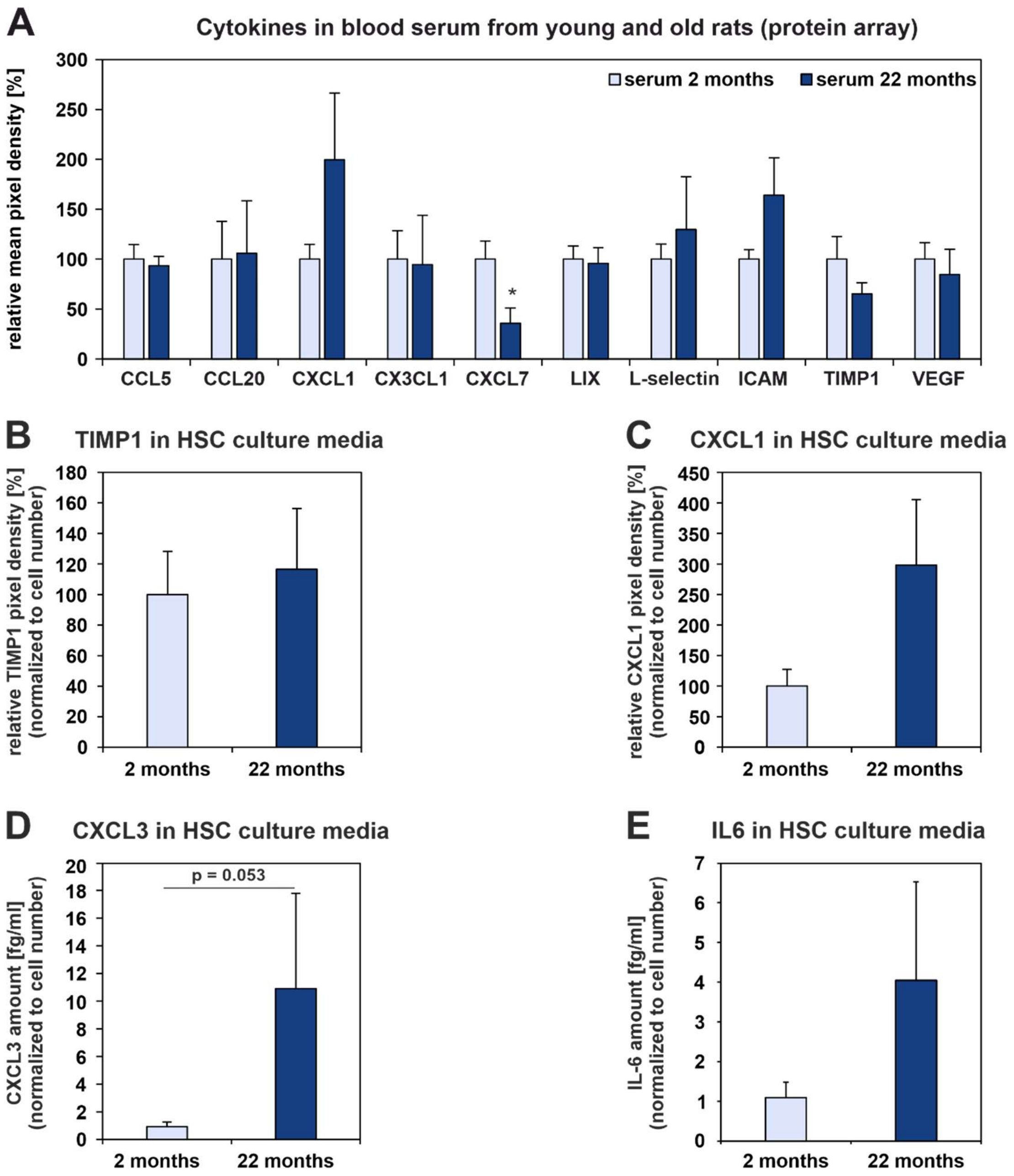

Supplemental Figure S4: Senescence- and inflammation-associated factors in blood serum and HSC. (A) The rat cytokine array revealed no obvious differences of cytokines in the serum of young and old rats, with the exception of CXCL7, which was significantly lower in the serum samples from 22-months-old animals. Mean pixel density of samples from 2-months-old rats was set to 100% and data are presented as means ± SEM (n = 4, *p < 0.05). Cytokines released into culture medium by freshly isolated HSC from young and old rats was also analyzed. Only TIMP1 and CXCL1 were detected by the rat cytokine array in all culture supernatants. **(B)** TIMP1 concentration remained unchanged, **(C)** whereas CXCL1 increased by 3-fold in culture supernatants of HSC from old rats (n = 4 for each age group). Data from young rats were set to 100%. **(D)** Analysis of CXCL3 by ELISA revealed a 10-fold increase in CXCL3 concentration in HSC culture media from old rats. **(E)** IL6 concentration in culture supernatants of HSC from old rats increased by 4-fold compared to young rats as analyzed by ELISA. Cytokine array and ELISA data were normalized to cell number. The data are presented as means ± SEM (cytokine arrays: n = 3; ELISA: n = 5 for 2-months and n = 3 for 22-months-old rats; *p < 0.05).

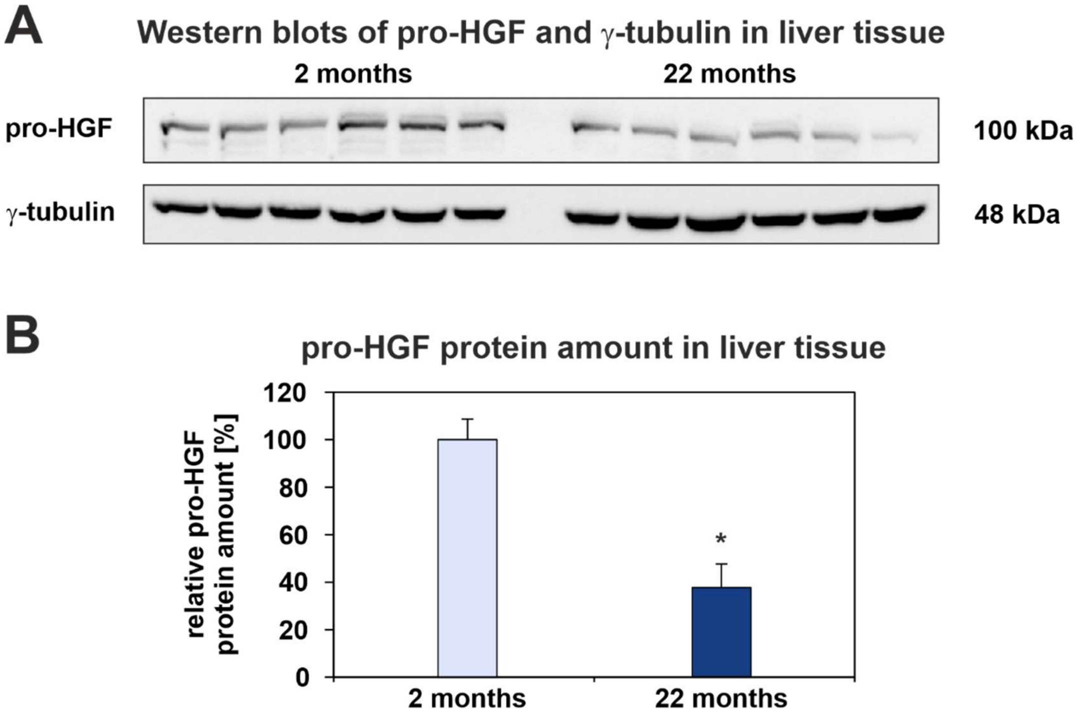

Supplemental Figure S5: Pro-HGF in rat liver tissue during aging. (G) Representative Western blot of pro-HGF in liver tissue from 2-months and 22-months-old rats. γ-Tubulin was used as loading control. **(H)** Western blot analyses of liver tissue from young and old rats revealed age-related significantly reduced pro-HGF amounts (n = 6 for each age group; * $p < 0.05$).

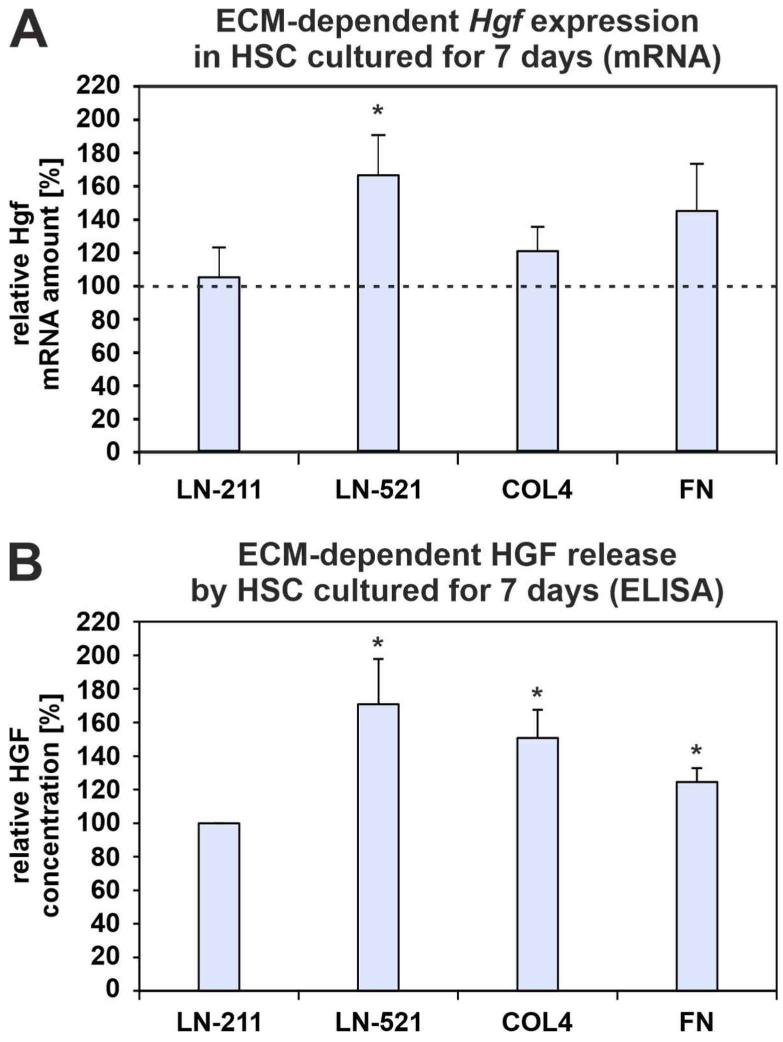

Supplemental Figure S6: Influence of ECM proteins on HGF expression and release by HSC. (A) Expression of *Hgf* in HSC was dependent on the ECM protein used for coating of culture dishes. HSC were cultured for 7 days under serum-free conditions on uncoated or ECM protein-coated (LN-211, LN-521, COL4, or FN) culture dishes. The mRNA expression of HSC cultured for one week on uncoated polystyrene dishes was set to 100% (broken line) for each experiment. The data are indicated as means ± SEM (n = 4-8; *p < 0.05). **(B)** HGF release by HSC from mid-aged rats cultured for one week on different ECM protein-coated surfaces was analyzed by ELISA and revealed that cells on LN-521, COL4, and FN released significantly more HGF than HSC cultured on LN-211. The HGF concentration measured for HSC on LN-211 was set to 100% for each independent experiment. The data are presented as means ± SEM (n = 4, *p < 0.05).
